# GTGO-driven joint task offloading and resource allocation with explainable AI in vehicular edge computing

**DOI:** 10.1371/journal.pone.0354977

**Published:** 2026-08-19

**Authors:** Aditi Moudgil, Shalli Rani, Fazlullah Khan

**Affiliations:** 1 Chitkara University Institute of Engineering and Technology, Chitkara University, Rajpura, Punjab, India; 2 Department of Computer Science, Faculty of Science and Engineering, University of Nottingham Ningbo China, Ningbo, Zhejiang, China; King Fahd University of Petroleum & Minerals, SAUDI ARABIA

## Abstract

Due to the fast development of intelligent transportation systems and connected vehicles, efficient computation offloading and resource management in vehicular edge computing (VEC) environments have become crucial issues. Low latency, optimality in resource usage, and clarity in decision-making is an open research issue. This paper presents a framework based on GTGO to jointly offload, schedule and allocate resources to different tasks and augment it with an integrated explainable AI (XAI) module. The proposed method enhances system welfare by approximately 15–25 percent and decreases the average task delay by 10–20 percent compared to the baseline approaches as the number of task vehicles increases. The GTGO algorithm converges rapidly and it will stabilize after 30–50 iterations hence guaranteeing computational efficiency. Also, the XAI module is a way of quantitatively understanding the contribution of decision variables to the interpretation of the results, without affecting optimization performance. These findings indicate that the suggested framework is an effective, efficient, and transparent resource management solution in intelligent vehicular edge computing systems.

## 1. Introduction

The future of the fully autonomous transportation system, with self-regulated traffic flow, little congestion, and greatly improved road safety, is gradually turning into a reality with the fast development of Autonomous Vehicles (AVs). These smart systems are based on an advanced system of sensing technologies that comprise cameras, Light Detection and Ranging (LiDAR), radar, and ultrasonic sensors to sense their immediate environment [1]. The information acquired with the help of these sensors is analyzed with the help of sophisticated Artificial Intelligence (AI) and deep learning algorithms that help vehicles to identify objects, identify trends, anticipate actions, and make real-time driving choices [2]. Although these are possible, onboard perception systems tend to be ineffective in real-world situations that are complicated, like in a dense urban setting, or blocked by large vehicles or infrastructure, or bad weather like fog, rain, or snow. These constraints make it clear that cooperative and distributed intelligence outside the vehicle is needed.

In order to address them, recent vehicles systems are becoming more and more dependent on communication paradigms like Vehicle-to-Vehicle (V2V), Vehicle-to-Infrastructure (V2I) and, more generally, Vehicle-to-Everything (V2X) communication [3]. These technologies allow vehicles to share important data on the road conditions, road hazards, pedestrian flow, and infrastructure signals, and therefore, provide them with a wider situational awareness than the sensor constraints of their local areas. These interrelated communication systems create dynamic wireless vehicular networks, upon which Intelligent Transportation Systems (ITS) are based. These networks help to create safer driving conditions, decrease traffic congestion and enhance transportation efficiency by allowing vehicles and infrastructure to coordinate their decisions [4].

Nevertheless, the growing trend of using data-driven decision-making in vehicular networks poses considerable computational and communication issues. The vast volume of disparate information that is delivered by the network of cars demands effective processing techniques that meet the high latency requirements, not least in the realm of software safety systems, such as collision avoidance, emergency braking and adaptive traffic control [5]. The conventional cloud-centric solutions that are based on centralized processing cannot be sufficient because of the high latency, bandwidth limitations, and possible network overload. In time sensitive vehicular conditions, where one millisecond can have a significant impact, such restrictions can also cause a delay in reaction time which may not be acceptable.

Here, Vehicular Edge Computing (VEC) and Edge AI have become paradigms shifting the computational resources nearer to the data source. VEC can be adopted by introducing edge servers at roadside units (RSUs) or base stations to decrease latency, lower bandwidth usage and improve the responsiveness of the system [6]. The edge AI is based on this approach with machine learning capabilities at the edge, which allows vehicles and surrounding physical objects to process and analyze information simultaneously in real time. This type of distributed intelligence model can be used in real-time object detection, prediction of traffic and cooperative driving strategies, as well as for improving the dependability and effectiveness of autonomous vehicles in general [7].

Yet there are still a number of significant challenges. Data privacy and security is one of the main issues. However, the traditional method of collecting massive amounts of vehicle data at central servers has high communication complexity and high risk of cyber attack and data leakage [8]. Furthermore, data sharing is subject to strict regulations like the General Data Protection Regulation (GDPR), which provide users with control over their personal data. Within vehicular networks, where information like the trajectory, driving behavior, or accident records are being generated in real time, safeguarding privacy becomes crucial. Furthermore, data owners might not be willing to provide all the data, resulting in incomplete data sets and potentially biased AI models [9].

The other critical drawback of the existing AI-powered car systems is the unavailability of transparency and interpretability. Although both deep learning and deep reinforcement learning (DRL) models have shown impressive results in tasks like traffic prediction, resource allocation, and autonomous navigation, they are commonly said to be black-box models as they have complex and opaque decision-making processes [10]. The lack of understanding and justification of model outputs becomes critical in safety-critical applications where choices may be life or death, and where there is a strong need to establish trust, accountability, and regulatory compliance. To give an example, when an autonomous car performs an emergency move, the interested parties – drivers, manufacturers, regulators, and so forth, should be capable of comprehending the logic behind the move [11].

Explainable Artificial Intelligence (XAI) fills this vital gap by offering interpretable information on AI model behavior [12]. The SHapley Additive exPlanations (SHAP), Local Interpretable Model-Agnostic Explanations (LIME), and Gradient-weighted Class Activation Mapping (Grad-CAM) methods allow visualizing and explaining model prediction, allowing users to determine the role of input features in the overall decision. Implementing the XAI in vehicle systems does not only help to increase transparency but also create user trust, debugging, and legal and ethical frameworks in the event of accidents or system failures.

More recent developments in wireless communication technologies (especially Cellular Vehicle-to-Everything (C-V2X)) enhance the opportunities of intelligent vehicular networks. C-V2X uses current cellular infrastructures to deliver high-bandwidth, low-latency, and reliable communication, even in high-mobility conditions [13]. New 6G networks will further increase these functions with the possibility to achieve ultra-reliable and low-latency communication (URLLC), massive connectivity, and intelligent network management. Nevertheless, the control of radio resources and Quality of Service (QoS) in these dynamic and complicated environments continues to be a difficult task [14]. Deep Reinforcement Learning (DRL) has emerged as a promising solution to solve these issues by helping to allocate resources adaptively and efficiently in high-dimensional state-action spaces. However, the unpredictability of DRL models restricts their use in safety-related vehicle systems.

The current challenges have motivated the development of a more promising combination of systems that can leverage the power of Edge AI, Federated Learning and Explainable AI to create intelligent, secure and trustful vehicle systems [15]. These frameworks are designed to support real-time decision-making and have low latency, keep data confidential and secure, and give interpretable information about model behavior. Such solutions can greatly enhance the safety, efficiency, and reliability of autonomous transportation systems by making it possible to conduct collaborative learning among distributed edge nodes without compromising transparency and accountability.

Thus, the current research aims to design a novel vehicular edge network architecture that combines explainable and AI techniques to enable intelligent transportation services. The recommended solution will take advantage of edge intelligence for ultra-low latency need and will use XAI techniques to ensure the interpretability and trust in decision-making. By extensive performance analysis based on performance metrics like accuracy, latency, throughput, and interpretability, this work seeks to make a contribution in the creation of next-generation intelligent transportation systems.

The reasons for this research are as follows:

- Due to the rapid advancement of connected and autonomous vehicles, there are more and more tasks that require minimum delay.In the context of connected and autonomous vehicles, the number of tasks requiring minimum delay has increased, thus task offloading and resource management (TOM) in vehicular edge computing (VEC) environments are becoming more and more challenging.
- The existing methods tend to focus on task offloading, scheduling, and resource allocation separately, leading to sub-optimal system performance and lower overall system welfare in varying network environments.
- Most of the optimization-based solutions are black-box solutions, namely with no interpretability and transparency, which makes them unreliable and not easily used in safety-critical vehicular applications.
- A unified optimization framework that can maximize over system welfare and that can incorporate explainable AI (XAI) for insight into decision making and more trust in intelligent edge systems is strongly needed.

Although there have been major developments in the field of vehicular edge computing, the current task offloading and resource scheduling solutions mainly focus on either latency or energy or resource consumption optimization without taking into account the combined effect of these on the overall system welfare. Additionally, many optimisation and deep learning based solutions are black box solutions, which offer little insight into why a decision is made to offload or allocate resources. This has a negative effect on user trust and makes the introduction of such methods difficult in safety critical areas in vehicles where accountability and explainability is key. To meet this requirement, this work introduces a new unified optimization framework called GTGO-XAI to jointly solve task offloading and resource allocation problems by integrating Explainable Artificial Intelligence (XAI) mechanisms. This framework combines GTGO with explainable decision-making, which ensures the system benefits both system welfare and latency and achieves maximum throughput while using resources efficiently, while at the same time offering clear explanations about the factors that influence the optimization decision making process, leading to greater trustworthiness and applicability in intelligent vehicular edge networks.

The uniqueness of this research stems from the design of the GTGO-XAI model that simultaneously handles task offloading, task scheduling, and resource allocation issues in Vehicular Edge Computing (VEC) along with providing an explanation for decision-making process. The difference between the proposed model and previous works is in the fact that current solutions do not handle the above mentioned tasks together and use black-box optimization methods. In contrast, the new approach combines an upgraded Genetic Task and Resource Governance Optimization (GTGO) algorithm and Explainable Artificial Intelligence (XAI) module in order to give understandable explanations of the decisions made during optimization. In addition, the proposed four-layer architecture allows for coordinated management of resources in vehicular, edge, intelligence, and control layers which leads to efficient utilization of computational resources under latency constraints. Simulation results show that the proposed GTGO-XAI model significantly increases system welfare, minimizes average task delay, quickly converges, and gives transparent explanations for decision-making process with a small amount of additional computation cost.

The remainder of this paper is organized as follows: The related work of task offloading, resource allocation and explainability in vehicular edge computing is summarized in Section II. In Section III, the system model is elaborated including the four-layer architecture, the utility functions of the task vehicles, the helper vehicles, and the controller as well as the problem formulation. In Section IV, the proposed GTGO algorithm and the XAI module are presented. Section V presents the simulation setup and experimental results with respect to system welfare, average task delay and convergence behavior. The paper is concluded in Section VI and directions for future work are shown.

## 2. Related work

Due to the fast development of intelligent vehicular networks, the privacy-preserving collaboration, edge intelligence, and AI-based decision-making have become the topic of extensive research [16]. Enabling distributed learning and efficient computation in autonomous vehicular environments has received a considerable amount of literature. Currently, privacy-conscious designs, especially federated learning, seek to minimize data sharing through centralized data distribution and instead enable vehicles to learn the models on-site and exchange updates only [17]. Although these methods are effective in dealing with privacy issues and reduce overhead in terms of computation at local edges, the majority of them primarily aim at reducing computation at the local edges or offloading tasks to edge resources that are underutilized. But they frequently miss one important point–that of relevance of transmissions of updates [18]. The importance of each locally generated update is not always equal to the model improvement in very dynamic vehicular situations, and it is necessary to judge their importance prior to transmission.

Local updates in the case of Autonomous Vehicles (AVs) are usually features derived based on sensory inputs that directly affect driving behavior, including the detection of objects, prediction of trajectory, and identification of hazards [19]. Although they are important, it is still a difficult task to define which features are most applicable because AI models at the edge have a rather complex nature. Recent car systems are based on deep learning models that consume real-time multimodal input (in the form of cameras, LiDAR, and radar) data. These models, despite being very precise, are opaque black boxes and it is not easy to explain how certain attributes contribute to a particular decision. The absence of transparency restricts the capability of maximizing the efficiency of communication and brings up issues of trust, accountability, and safety in life-threatening driving situations [20].

Vehicular Edge Computing (VEC) has gained a lot of popularity as an answer to high latency and computational needs of smart transportation systems. VEC allows the real-time use of computationally intensive applications like collision detection, traffic prediction, and video based monitoring by taking computation nearer to the source of the information [21,22]. It has been shown in previous research that the combination of deep learning and edge computing can greatly improve the responsiveness of a system and its bandwidth utilization [23]. Nevertheless, these papers mainly focus on performance measures like latency, throughput and accuracy, which frequently overlook the interpretability of underlying models. Consequently, despite its practical success, the explainability of these systems is a drawback that impedes their use in safety-critical processes where it is important to know the reason behind the decisions taken.

Recent studies have deployed Explainable Artificial Intelligence (XAI) methods into vehicular and edge computing systems to deal with the interpretability challenge [24]. XAI approaches, such as SHapley Additive exPlanations (SHAP), Local Interpretable Model-agnostic Explanations (LIME), Permutation Feature Importance, and Partial Dependence Plots (PDP) have been used to identify feature contributions and have been used to give insights into model behavior. Such methods allow the stakeholders to know the reasons why a specific decision was made, where the decision may break down, and how the individual features read into predictions [25]. XAI integration has demonstrated potential in enhancing transparency and ease of debugging systems as well as increasing user trust in vehicular networks. Moreover, feature selection methods based on XAI can be used to reduce the less meaningful updates, and, therefore, unwanted communication and computational costs in edge networks [26].

Recent studies have also been interested in the convergence of federated learning and XAI. The federated learning allows multiple vehicles to cooperatively train models without exchanging raw data, which is appropriate in situations that demand privacy. It can be used in conjunction with XAI to give an extra degree of interpretability, enabling distributed models to produce both explanable insights and predictions [27]. Other papers have discussed the application of XAI measures, including trust scores based on feature importance, to inform the participant selection and aggregation in federated learning systems [28]. Also, other methods like differential privacy and blockchain have been suggested to increase the level of security, transparency and trust in collaborative vehicular systems [12]. Although all these developments have been made, none of the available solutions address privacy preservation and interpretability in isolation, and there is a little effort made on their combined application in real-time vehicles.

Resource allocation is one of the main issues in the field of vehicular communications, especially Cellular Vehicle-to-Everything (C-V2X) networks. Conventional centralized methods are based on channel state information on a global channel and optimization iteratively, which in dynamic and large scale vehicular settings is typically not feasible because of the computational complexity and communication cost [29]. To defeat these drawbacks, Deep Reinforcement Learning (DRL) is becoming more popular in resource management. DRL-based procedures can operate with high-dimensional state-action areas and adjust to changing network circumstances. Nevertheless, like any other deep learning method, DRL models lack a sense of explanations, so their decision-making mechanisms are hard to understand [30]. This is a major constraint especially in safety-sensitive applications where the reasons behind the decision to allocate resources are vital.

Recent works have tried to integrate XAI to wireless communication systems to cope with the performanceexplainability trade-off. The importance of features in network slicing, intrusion detection and resource allocation have been studied with techniques like SHAP. These techniques are a great source of information on how the models behave and assist in determining the main determinants of decisions. The majority of existing works, however, are aimed at post-hoc explanation as opposed to an explicit inclusion of explainability in the decision-making pipeline. Furthermore, feature selection algorithms based on XAI can be computationally expensive and they might be inappropriate in resource constrained edge case scenarios. Furthermore, most of the proposed explainability metrics have close connections with particular algorithms, which restricts their applicability to different models and uses [31].

Table 1 briefly reviews selected research on vehicular edge computing, federated learning, explainable artificial intelligence, and intelligent resource allocation. One can notice that the existing works are mainly oriented towards one of the two aspects, namely performance optimization and interpretability, separately. There are very few studies that explain the task offloading problem, resource allocation problem and the problem of explainable decision making in one framework. This constraint is the motivation for developing the proposed GTGO-XAI framework that combines intelligent optimization and explainable resource management for vehicular edge computing environments.

**Table 1:** Comparison of Existing Approaches and the Proposed GTGO-XAI Framework.

Reference	Optimization Technique	Explainability	Advantages	Limitations
[17,18]	Federated Learning	None	Privacy-preserving distributed learning	Limited interpretability and no joint resource allocation
[21–23]	Vehicular Edge Computing	None	Low latency and improved computation efficiency	Focuses mainly on performance metrics
[24–26]	XAI-based Methods	SHAP, LIME, PDP	Improved interpretability and trust	Limited optimization capability
[12,27,28]	Federated Learning + XAI	Feature Importance	Privacy preservation and explainability	Limited support for real-time VEC
[29,30]	DRL	Limited/Post-hoc	Adaptive resource allocation	Black-box decision making
[31]	XAI-enabled Wireless Systems	SHAP	Explains resource allocation decisions	Computational overhead
[32]	Reinforcement Learning	Not Explicit	Adaptive privacy-aware optimization	Focused on healthcare systems
**Proposed GTGO-XAI**	**GTGO**	**XAI-based Feature Analysis**	**Joint offloading, resource allocation and interpretability**	**Moderate computational overhead**

Reinforcement learning is gaining more and more popularity recently due to its applications in intelligent decision-making, privacy preservation and adaptive optimisation in distributed systems. In digital healthcare systems, for instance, the RLDJ-W framework proposed by [32] integrates the reinforcement learning (RL) approach to synergistically design watermarking strategies for privacy leakage detection (PLD). The framework provides a dynamic method to adapt to changing conditions in the system, meeting security and performance goals through learning-based approaches. While RLDJ-W is applicable to resource management issues in healthcare systems, the concept of adaptive decision making and intelligent optimization is closely related to resource management in vehicular edge computing. On the other hand, the proposed GTGO framework is aimed at task offloading and resource allocation in vehicular network, with additional explainable decision making mechanisms to enhance the transparency and trustworthiness of the system. Finally, as evidenced by the success of reinforcement learning-based systems, like the RLDJ-W, there is a need for intelligent optimization methods in next-generation distributed computing environments.

Although tremendous advances have been made in vehicular edge computing, there are still some challenges in current vehicular task offloading and allocation strategies. The existing methods mostly concentrate on minimizing the latency, energy usage or resource utilization, and regard the task offloading and the resource allocation as two independent optimization problems. Moreover, numerous recent intelligent optimization frameworks use a black-box decision making process that do not give much insight into the reasoning behind the offloading and resource allocation decisions. This uncertainty of interpretation can make users less trustful and make it difficult to implement such solutions in safety critical vehicle applications.

Moreover, approaches that combine explainability with optimization in a unified framework are scarce in the literature. Explainable Artificial Intelligence (XAI) has shown great promise in enhancing the transparency and accountability in intelligent systems, but its use in vehicular Edge Computing resource management is still under-researched. Therefore, an optimization framework that can optimize task offloading and resource allocation while interpreting the decisions and ensuring high system performance is lacking in the research literature.

In order to tackle these challenges, this paper introduces the GTGO-XAI framework that combines two key elements: Genetic Task Generation Optimization (GTGO) and Explainable Artificial Intelligence (XAI) for intelligent task offloading and resource allocation in VEC. The proposed framework jointly optimizes the offloading decisions and resource allocation decisions, and at the same time gives interpretable explanation of the decisions it makes. With this integration, GTGO-XAI adds to the benefit of the entire system, decreasing task delay, improving transparency and trustworthiness in the vehicular edge computing system and improving the welfare of the system.

In general, the literature reflects the considerable advancement in the development of intelligent, privacy-concerned, and efficient vehicular systems. However, there are quite a number of essential gaps. To begin with, the collaborative vehicular learning frameworks lack the mechanisms to assess and communicate only pertinent updates. Second, the current AI models on the edge do not have an essential interpretability, which is fundamental to the safety-critical decision-making. Third, although XAI methods offer important insights, their combination with real-time edge systems is still limited by the computational resource requirements. Lastly, lack of coherent frameworks that all consider privacy, efficiency, and explainability in vehicular networks exist [33].

In order to fill these gaps, new methods that utilize XAI to assess feature relevance at the edge are urgently needed to selectively transmit valuable updates with low latency and high accuracy. These methods can greatly improve the efficiency, transparency, and credibility of AI-powered vehicular systems, leading to the creation of safer and more reliable intelligent transportation networks.

## 3. System architecture

**Fig 1:**
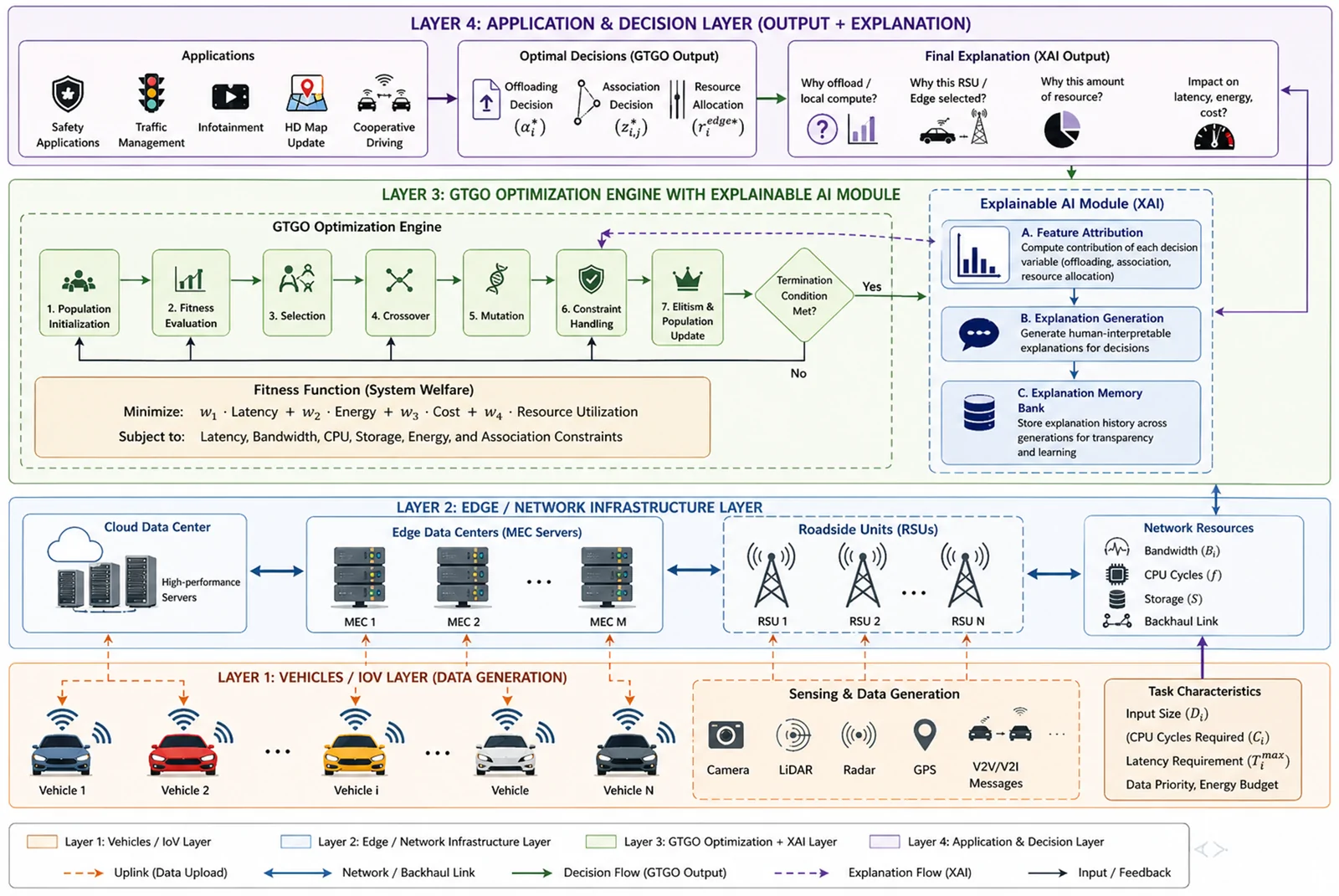
Proposed System Architecture.

Fig 1 shows the four layer architecture consisting of The layers of the vehicle are divided into vehicular layer, edge layer, intelligence & explainability layer and the control layer. Below are some summaries of how each layer works.

1. **Vehicular Layer:** The vehicular layer consists of: Task vehicles and Helper vehicles, which are the computing entities in the system. The task vehicles generate temporal computational tasks with characteristics that include input data size $S_{i}$, computational demand $C_{i}$ and delay threshold $\tau_{i}^{lim}$. The tasks may be performed locally based on the status of the system and optimization results, or they may be partially offloaded. All available computational resources $\psi_{j}$ of helper vehicles are used to help offload tasks. Offloading fraction $\alpha_{i}$ and task assignment $z_{i,j}$ are not determined locally but are coordinated by the proposed framework, as part of a system-wide optimization.
2. **Edge Layer:** The edge layer consists of Road Side Units (RSUs) with extra computing power. If local resources/ helper resources are inadequate/ suboptimal, RSUs are used to execute tasks. The $r_{i}^{edge}$ resource allocation on the edge is calculated as an outcome of the joint optimization process explained in the methodology. This layer serves as an intermediate processing layer which allows for low latency computation, and also helps to achieve the overall system goal of maximizing social welfare.
  The Intelligence and Explainability Layer is characterized by two components.The Intelligence and Explainability Layer consists of two parts. The proposed system’s decision making body is the intelligence and explainability layer. It uses the Genetic Optimization Engine (GTGO) for jointly optimizing the decision variables of offloading ratio $\alpha_{i}$ of tasks, allocation $z_{i,j}$, and resource allocation $r_{i}^{edge}$, as discussed in the system optimization problem. The goal is to maximize the overall system welfare 𝒲, under delay and resource constraints, following the methodology.
  This layer also includes an explainability module that offers interpretable explanations of the decision-making process, in addition to optimization. An explanation function ${ℰ}_{i}$ is created for every task vehicle *i* to explain why some factors like task size, computational requirements, delay threshold $\tau_{i}^{lim}$, and resources availability, etc influence the final decisions. In addition, the module decomposes the welfare of the system to illustrate trade-offs between delay, costs, and resource use. This integration guarantees the optimization process is streamlined and clear, matching the decision explanation step in the system process.
3. **Control Layer:** The control layer is in charge of system coordination and decision making. It extracts the information of the system state, which is represented by $\Omega_{i}$ and $\Psi_{j}$ respectively, from the vehicle layer, and designs its incentive mechanism by calculating the price of the service provided by the task vehicle $\pi_{i}$ and the reward given to the helper vehicle $\gamma_{j}$. These are used to control the behaviour of the system entities, as well as inputs to the optimization problem.
  The control layer communicates with the intelligence layer via the exchange of information on the state of the system and the receipt of optimized decisions $\left\{\alpha_{i}^{*},z_{i,j}^{*},r_{i}^{edge*}\right\}$. It then propagates these decisions throughout the network, coordinates the task execution and controls incentive transactions. This corresponds to the system state acquisition, decision implementation and system update steps in the workflow, thus facilitating coordinated operation of the system.
  In general, the workflow starts with the generation of tasks at the vehicular layer, followed by collection of the resources at the edge layer. The collected system state information is fed to the GTGO optimization engine to optimize offloading and resources allocation decisions. The XAI module then produces understandable explanations of those decisions, which are then passed on and applied across the network by the control layer. Table 2 summarizes all the notations and symbols used for detailing of the proposed GTGO approach.

**Table 2:** Summary of Notations Used in the Proposed GTGO-XAI Framework.

Symbol	Description
*i*	Index of task vehicle
*j*	Index of helper vehicle
$S_{i}$	Input data size of task *i* (MB)
$C_{i}$	Computational demand of task *i* (MIPS)
$\tau_{i}^{lim}$	Delay threshold (maximum allowable delay) of task *i* (s)
$\alpha_{i}$	Task offloading ratio of task *i*
$z_{i,j}$	Task assignment variable indicating allocation of task *i* to helper vehicle *j*
$r_{i}^{edge}$	Edge resource allocation assigned to task *i*
$\psi_{j}$	Available computational resources of helper vehicle *j* (MIPS)
$\Omega_{i}$	State information of task vehicle *i*
$\Psi_{j}$	State information of helper vehicle *j*
$\pi_{i}$	Service price associated with task vehicle *i*
$\gamma_{j}$	Incentive/reward allocated to helper vehicle *j*
$U_{i}$	Utility of task vehicle *i*
$U_{j}$	Utility of helper vehicle *j*
$U_{mgr}$	Utility of the system controller/manager
*W*	Overall system welfare
$E_{i}$	Explainability score/explanation function for task vehicle *i*

## 4. Proposed methodology

### 4.1. System model overview

The suggested system takes into account a vehicular edge computing system comprising of task vehicles, helper vehicles, and edge nodes that are coordinated by a centralized controller. Every task vehicle creates delay-sensitive computation tasks based on the input data size, computational requirements, local processing capability, and a threshold. The helper vehicles also contribute the idle computational resources to help in executing the tasks, and the edge nodes also contribute to offering further computing support where necessary. The system tries to combine the optimization of offloading of tasks, scheduling, and allocation of resources in a way that maximizes the system welfare as a whole and meets the delay constraints.

### 4.2. Computation and communication model

The communication and computation model defines the behavior of data transmission and processing of the system. The rate at which information is transmitted between task vehicles and helper vehicles is determined by the conditions and bandwidth of the channel. The total task delay is the sum of the local computation delay, transmission delay of the offloaded part, and the implementation delay on the chosen computing node. In particular, the work is done locally in a part and offloaded to either helper cars or edge nodes in the other part. The maximum of the local processing delay and offloaded processing delay is defined as the total task completion time since it is important that the final delay is the actual delay in which the entire task is completed. This model is a foundation of the assessment of system performance in varied offloading choices.

### 4.3 Utility Functions

In order to measure the performance of the system, utility functions are established with respect to all the entities involved. The utility of task vehicles is an indication of the trade-off between reduce delay and service cost, the lower the delay compared to the threshold, the better the user satisfaction, and the higher the offloading, the higher the cost. The incentives available to provide computational resources is matched against the cost of using resources to determine the utility of helper vehicles. The utility of the controller takes into consideration the income of the task vehicles and the incentives paid to helper vehicles, as well as the expense of using edge resources. These individual utility functions are summed up to give the total system welfare that is the optimization target of the given framework.

Equation (1) represents the utility of a task vehicle as the trade-off between the benefit gained from reduced task completion delay (captured using a logarithmic function) and the cost incurred due to task offloading and resource consumption.


$$\begin{array}{c} U_{i}=\eta_{i}\ln\left(1+\lambda_{i}\left(t_{i}^{lim}-T_{i}^{fin}\right)\right)-\pi_{i}\alpha_{i}S_{i} \end{array}$$ (1)

Equation (2) represents the utility of a helper vehicle as the difference between the incentive received for cooperation and the cost associated with resource usage, modeled as a quadratic penalty.


$$\begin{array}{c} U_{j}=\gamma_{j}-\xi \psi_{j}^{2} \end{array}$$ (2)

Equation (3) represents the utility of the controller as the net revenue obtained from task offloading payments minus the incentives paid to helper vehicles and the cost of edge resource utilization.


$$\begin{array}{c} U_{mgr}=\sum_{i}\pi_{i}\alpha_{i}S_{i}-\sum_{j}\gamma_{j}-\zeta \sum_{i}r_{i}^{edge} \end{array}$$ (3)

Equation (4) represents the overall system welfare as the aggregate utility of all task vehicles, helper vehicles, and the controller.


$$\begin{array}{c} 𝒲=\sum_{i}U_{i}+\sum_{j}U_{j}+U_{mgr} \end{array}$$ (4)

### 4.4. Problem formulation

System optimization problem is defined as a joint maximization of the overall system welfare through finding the optimal offloading decisions, task assignment and resource allocation. The decision variables will be the offloading fraction and tasks assignment to helper vehicles, and edge resource allocation. There are many constraints to the optimization including delay constraints that make sure that the time of task completion is not above the threshold, assignment constraints that make sure that a task is not assigned to a particular user more than once, resource constraints that provide the maximum available edge capacity. The problem is complex due to the combination of continuous and discrete variables, and nonlinear relationships, which makes it difficult to solve with the traditional methods of optimization. The optimization objective maximizes the system welfare $\max_{\alpha_{i},z_{i,j},r_{i}^{edge}}𝒲$ subject to latency constraints $T_{i}^{fin}\leq \tau_{i}^{lim}$, bounded offloading decisions $0\leq \alpha_{i}\leq 1$, binary task assignment $z_{i,j}\in \{0,1\}$ with $\sum_{j}z_{i,j}\leq 1$, and limited edge resources $\sum_{i}r_{i}^{edge}\leq R^{max}$.

### 4.5. GTGO-based optimization framework

A Genetic Optimization-based solution (GTGO) is suggested to effectively address the developed optimization problem. The offloading fraction, task assignment, and resource allocation decisions are represented as a chromosome in this framework, so each candidate solution has a chromosome. The quality of each solution is determined by a fitness function that depends on the system welfare with penalty terms to impose a constraint satisfaction. The optimization is carried out in iterative steps of evolving the population of solutions by use of selection, crossover, mutation and elitism. It evolves a solution space that has the ability to effectively search for the solution, converge to near-optimum solutions without gradient information or extensive training and can be used in dynamic vehicular environments. The decision variables are task offloading, task assignment, and edge resource allocation and are included in the decision vector 𝐗, as shown in equation (5).

**Algorithm 1 GTGO-Based Joint Offloading and Resource Optimization with XAI Module**

**Require:** System state $\Omega_{i},\Psi_{j}$, pricing $\pi_{i}$, incentives $\gamma_{j}$

**Ensure:** Optimal decisions $\left(\alpha_{i}^{*},z_{i,j}^{*},r_{i}^{\text{edge}*}\right)$ and explanation ${ℰ}_{i}$

1: Initialize population ${𝒫}^{\left(0\right)}$ with random chromosomes $𝐗=\{\alpha_{i},z_{i,j},r_{i}^{\text{edge}}\}$

2: Initialize XAI module ${ℳ}_{\text{XAI}}$ and explainability history buffer ℬ←∅

3: Set generation counter g←0

4: **while** termination condition not satisfied **do**

5:   Evaluate fitness ℱ(𝐗) for each chromosome based on system welfare *W*

6:   Compute per-variable contribution scores $𝒮(x_{m})$ via ${ℳ}_{\text{XAI}}$

7:   Aggregate weighted explainability score $\Phi_{k}$ from contribution scores $𝒮(x_{m})$

8:   Store $\Phi_{k}$ in explainability history: $ℬ\leftarrow ℬ\cup \{\Phi_{k}\}$

9:   Select parent chromosomes using fitness-proportionate selection

10:  Apply crossover with rate $p_{c}$ to produce offspring ${𝐗}^{\text{off}}$

11:  Apply Gaussian mutation with rate $p_{m}$ to introduce diversity

12:  Repair any infeasible offspring; apply penalty to fitness for constraint violations

13:  Form next generation via elitism: ${𝒫}^{\left(g+1\right)}\leftarrow {𝒫}_{\text{elite}}\cup {𝒫}_{\text{offspring}}$

14:  g←g+1

15: **end while**

16: Select optimal chromosome: $\left(\alpha_{i}^{*},z_{i,j}^{*},r_{i}^{\text{edge}*})\leftarrow \text{arg}\max_{𝐗\in 𝒫}\;ℱ(𝐗\right)$

17: Generate decision explanation: ${ℰ}_{i}\leftarrow {ℳ}_{\text{XAI}}(\alpha_{i}^{*},z_{i,j}^{*},r_{i}^{\text{edge}*})$

18: **return** $\left(\alpha_{i}^{*},z_{i,j}^{*},r_{i}^{\text{edge}*}\right)$ and ${ℰ}_{i}$


$$\begin{array}{c} 𝐗=\{\alpha_{i},z_{i,j},r_{i}^{edge}\} \end{array}$$ (5)

$$\begin{array}{c} \text{Fitness}(𝐗)=𝒲-\lambda \cdot \text{Penalty} \end{array}$$ (6)

The fitness function equation (6) quantifies a solution by maximizing system welfare, with the aim of penalizing any constraint violation.

#### 4.5.1. Algorithmic Innovations of GTGO.

The Genetic Algorithms (GA) have been used in many optimization problems, standard GA techniques do not address the dynamically changing and constrained conditions of vehicular edge computing environments. The proposed Genetic Task and Resource Governance Optimization (GTGO) framework is a modification of the traditional GA, focusing on introducing several task-oriented optimization mechanisms to enhance the convergence efficiency, quality of interpretability.

1. JMCE: Joint Multi-Dimensional Chromosome Encoding GTGO encodes task offloading decisions, helper vehicle assignment decisions, and edge resource allocation decisions in a single chromosome as opposed to optimizing a single decision variable in conventional GA implementations:

$$\begin{array}{c} X=\{\alpha_{i},z_{i,j},r_{i}^{edge}\} \end{array}$$ (7)

where $\alpha_{i}$ denotes the offloading ratio, $z_{i,j}$ represents task assignment decisions, and $r_{i}^{edge}$ represents allocated edge resources. It facilitates GTGO to collectively optimize interdependent decision variables, not optimizing them separately, and hence increase overall system welfare.

2. Constraint-Aware Fitness Evaluation: Typical GA implementations only consider the objective function values when determining fitness. On the other hand, GTGO also considers constraints such as latency, assignment and resource constraints directly in the fitness function using adaptive penalty mechanisms:

$$\begin{array}{c} F(X)=W-\lambda P(X) \end{array}$$ (8)

where *W* denotes the overall system welfare, λ is the penalty coefficient.

In the proposed framework, explainability is enabled by a mechanism that quantifies the influence of the key system parameters on the optimization decisions: feature contribution analysis. The features considered are the size of the tasks that needs to be input ($S_{i}$), the computational demand of the task ($C_{i}$), the delay threshold ($\tau_{i}^{lim}$), the resources of the helper vehicles ($\psi_{j}$), the offloading ratio of the task ($\alpha_{i}$), and the allocated edge resources ($r_{i}^{edge}$).

A contribution score $S(x_{m})$ is calculated for each of the optimized solutions, where it is measured based on the contribution of every decision variable $x_{m}$ to the overall system welfare. The contribution score is higher the more influence it has on the final decision of offloading and allocating resources.

The explainability score is then computed with Equation (10) where the weight of each feature is its importance. The resulting score is an interpretable representation of factors that contribute to optimization outcomes, and can be used to inform stakeholders about the trade-offs between delay, resource use and system welfare.

The explainability module is not a part of the optimization process; rather, it is a post-optimization analysis module. Rather, it works to provide transparency and insight into the most powerful decision influencing factors of decisions made with the GTGO framework.


$$\begin{array}{c} P(X)=P_{\text{delay}}+P_{\text{resource}}+P_{\text{assignment}} \end{array}$$ (9)

represents the aggregated constraint violation penalty associated with latency, resource allocation, and task assignment constraints, respectively.

3. Explainability-Guided Decision Assessment The key feature of GTGO is the incorporation of explainability information into its optimization framework. The XAI module produces feature contribution scores that assess how different features contribute to the optimization results based on the size of the task, the workload, the delay threshold and the availability of resources. Each solution is given an explainability score as:

$$\begin{array}{c} \Phi_{k}=\sum_{m=1}^{M}w_{m}S(x_{m}) \end{array}$$ (10)

where $S(x_{m})$ denotes the contribution score associated with decision variable $x_{m}$, and $w_{m}$ represents its corresponding importance weight. The aggregated explainability score $\Phi_{k}$ represents the overall contribution of the optimization variables to the final resource allocation and task offloading decisions, thereby enhancing the transparency of the proposed GTGO framework.

### 4.6. Explainability model

An explainability module is added to the proposed framework to increase transparency and interpretability. The paper in this module examines how the key system parameters, which include task size, computational demand, threshold and resource availability, impact the ultimate optimization decisions. An explanation function is created to explain the reasoning behind the choice of a specific offloading strategy, assignment of tasks, or allocation of resources, etc. Also, the model breaks down system overall welfare into the individual parts to indicate the trade-offs between delay, cost, and resource usage. This helps improve the comprehension of system behavior and increases confidence in the decision-making process which is a main weakness of black-box approaches. An explainability module is added to the framework to increase transparency.


$$\begin{array}{c} \varepsilon_{i}=f\left(s_{i},c_{i},\phi_{i},\psi_{j},l_{i}^{\text{lim}},a_{i},z_{i,j},r_{i}^{\text{edge}}\right) \end{array}$$ (11)

Equation (7) defines the explanation function $\varepsilon_{i}$ as a mapping of multiple system and decision-related parameters.


$$\begin{array}{c} \varepsilon_{i}=\sum_{k}\omega_{k}F_{k} \end{array}$$ (12)

Equation (8) expresses $\varepsilon_{i}$ as a weighted sum of contributing features, where $\omega_{k}$ represents the importance of each factor $F_{k}$.


$$\begin{array}{c} W=\sum_{i}u_{i}+\sum_{j}u_{j}+u_{mgr} \end{array}$$ (13)

Equation (9) represents total welfare *W* as the combined utility of all entities indexed by *i* and *j*, along with the manager.

#### 4.6.1. Computational complexity analysis.

The computational complexity of GTGO framework is shown by Equation (14).


$$\begin{array}{c} T_{GTGO}=O(GPN) \end{array}$$ (14)

where *G* denotes the number of generations, *P* represents the population size, and *N* is the number of task vehicles.

Fitness evaluation is carried out for each chromosome over many generations, resulting in a linear increase in complexity as the number of task vehicles and population size increase. This scalability allows GTGO to be suitable for the dynamic vehicular edge computing environment. Moreover, based on the results of the convergence, the best solutions are usually achieved after 30–50 generations, making the algorithm very usable for scenarios that require the deployment of the algorithm in real-time, with a latency requirement.

## 5. Results and discussion

All the simulation results after the performance evaluation of the proposed approach GTGO- vehicular based edge computing framework are presented in this section. The variations were made in the number of task vehicles for conducting the experimentation and comparisons were made across three already existing algorithms- Greedy offloading, Local-Only Processing, and Random Assignment. Metrices like system welfare, mean task delays, convergence optimization, feature-level explainability, and welfare distribution among other system entities.

### 5.1. System simulation setup

The simulations model a vehicular edge computing environment with N task vehicles (ranging from 5 to 30), 10 helper vehicles, and RSU-based edge nodes managed by a central controller. Task inputs vary per vehicle: data size $S_{i}$ between 0.5 and 5 MB, computational load $C_{i}$ between 100 and 500 MIPS, local processing capacity $\phi_{i}$ between 100 and 300 MIPS, and a delay threshold $\tau_{i}^{lim}$ between 1.0 and 2.0 s — all drawn from uniform distributions to capture the heterogeneity typical of real traffic scenarios. Helper vehicles offer idle resources $\psi_{j}$ up to 800 MIPS; the edge cap sits at $R_{\max}$ = 1000 MIPS. The cost coefficients ξ = 0.01 (helper resource) and ζ= 0.02 (edge resource) were set conservatively so that helper and controller utilities stay positive across all load conditions — pushing these higher collapses participation. For GTGO, a population of 50 chromosomes evolves over 100 generations, with crossover rate 0.8, adaptive mutation at 0.1, and penalty weight λ = 10 to keep infeasible solutions out of the running. Results are averaged over 50 independent runs as shown in 3.

**Fig 2:**
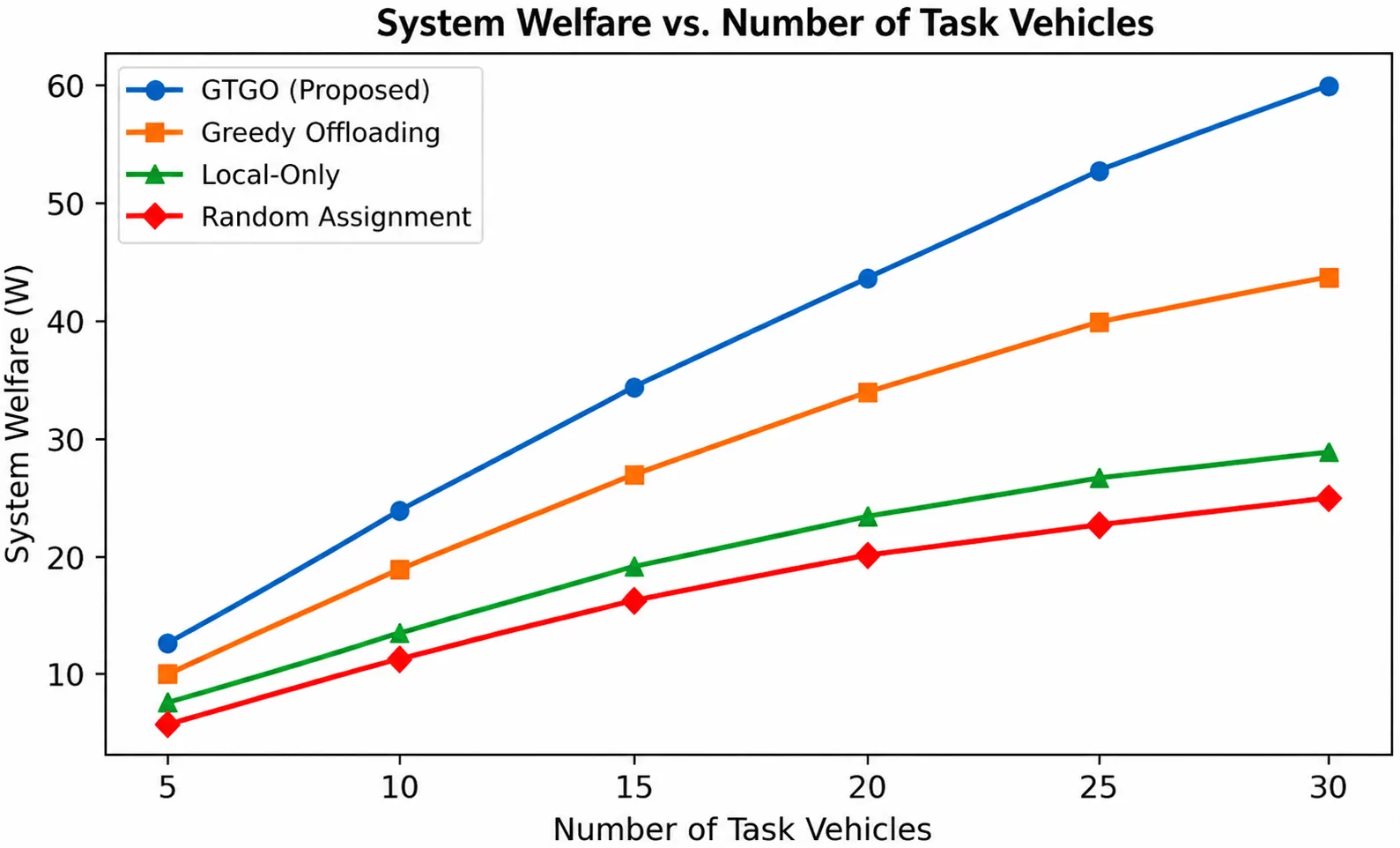
System Welfare vs number of task vehicles.

As shown in Fig 2 the system welfare for the proposed approach GTGO is directly proportional to number of task vehicles growing from 5–30. When GTGO is compared with all the three baseline models, the proposed approach yields 59.8 at N = 30 which is the maximum system welfare and proves that it is better than the other three models. GTGO jointly optimizes offloading fraction, task assignment, and edge resource allocation in one pass, rather than handling each decision independently (Table 3).

**Table 3:** Simulation Parameters.

Parameter	Value / Range	Description
Number of Task Vehicles (*N*)	5–30	Varied for scalability analysis
Task Input Data Size ($S_{i}$)	0.5–5 MB	Uniformly distributed
Computational Requirement ($C_{i}$)	100–500 MIPS	Task complexity metric
Delay Threshold ($\tau_{i}^{lim}$)	1.0–2.0 s	QoS latency constraint
Helper Vehicle Resources ($\psi_{j}$)	200–800 MIPS	Idle CPU capacity
Max Edge Resource ($R_{\max}$)	1000 MIPS	RSU edge capacity limit
GTGO Population Size	50	Chromosomes per generation
GTGO Max Generations	100	Convergence limit

**Fig 3:**
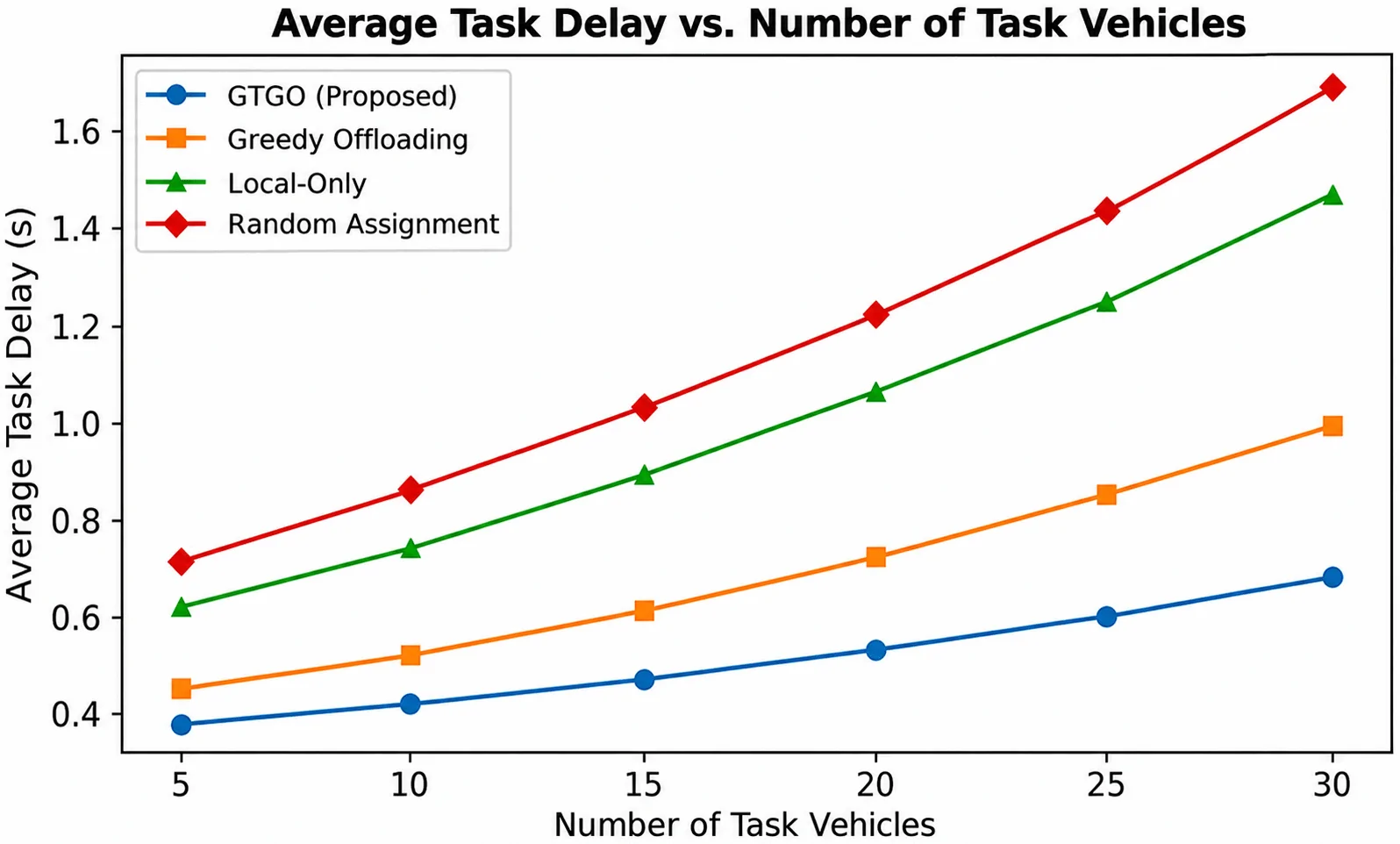
Average Task Delay vs number of task vehicles.

Fig 3 shows the comparison of average task delay and number of task vehicles. The minimum delay across all vehicle counts is shown by GTGO, i.e.,: 0.68 s when N = 30. In contrast, the local only delay algorithm degrades the processing sharply to 1.47 s with increase in the vehicle load. The greedy algorithm shows a moderate performance but GTGO consistently outperforms it.

**Fig 4:**
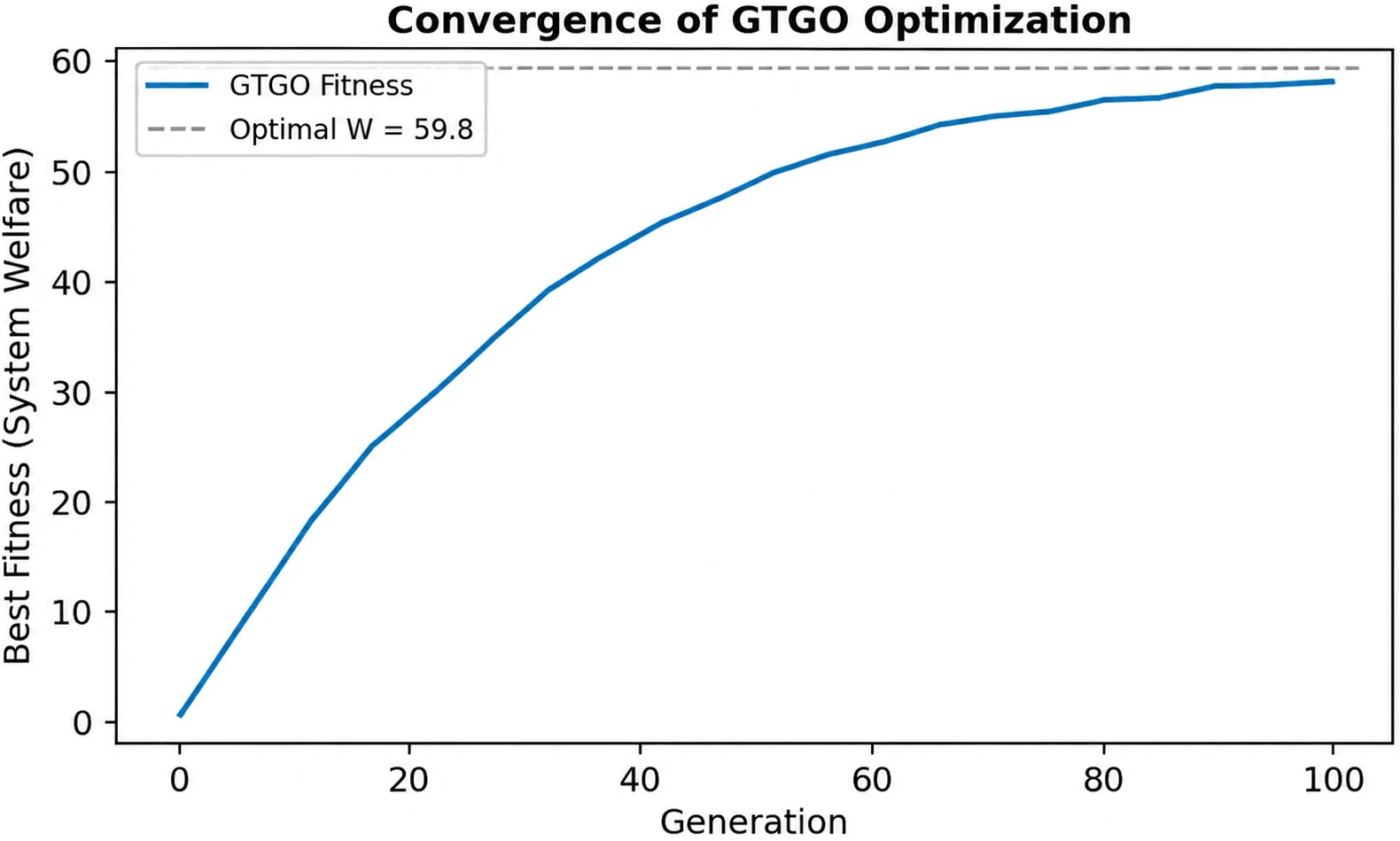
Convergence of GTGO Optimization (N = 30).

Fig 4 shows the convergence curve after implementing GTGO algorithm where N = 30 (number of task vehicles). The value of system welfare gains a steep rise for first 25 generations, after that it becomes stabilized for optimal value of 59.8 by generation 60. The fast convergence suggests that the evolutionary steps—selection, crossover, adaptive mutation, and elitism—work well together, balancing the search between exploring new possibilities and refining the best solutions within the mixed-integer space. As per the graph, no improvements were shown after generation 70.

The experimental results show that the proposed approach GTGO along with a 4- layer system architecture outperforms the baseline methods when evaluated on the evaluation metrics. These results validate both the effectiveness and the interpretability of the proposed framework for intelligent vehicular edge computing.

### 5.2. Performance analysis and trade-offs

**Fig 5:**
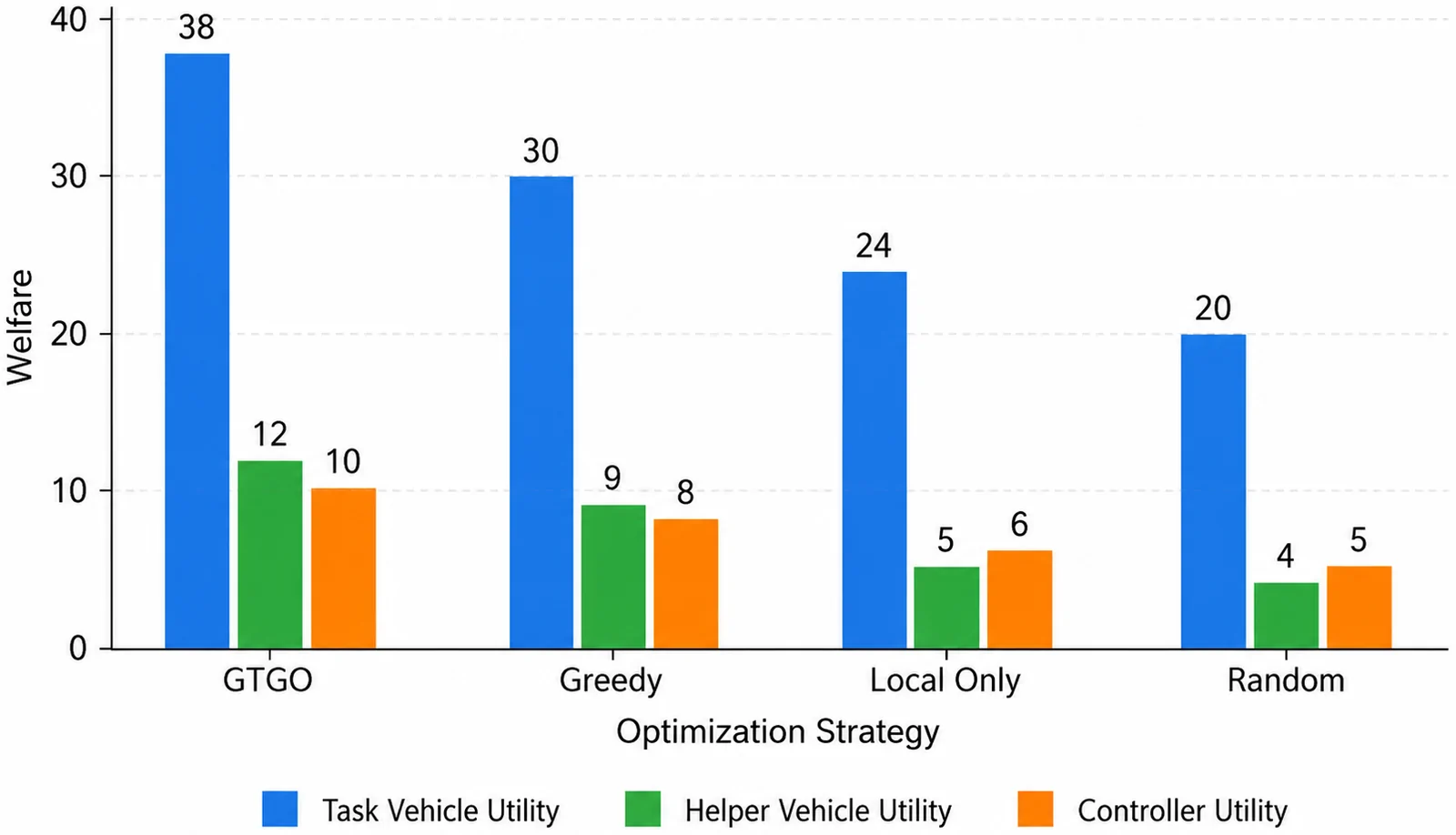
System Welfare Component Breakdown (N = 30).

The welfare gain that is obtained by GTGO is explained in the welfare breakdown displayed in Fig 5. The proposed scheme will increase the utility for the task vehicles in terms of decreasing task completion time and better offloading decisions. Meanwhile, the helper vehicles become more useful because computational resources are better utilized, and the controller is better equipped to handle resources and less inefficient in terms of resource allocation. Compared with the baseline approaches, where only partial performance is optimized, GTGO optimizes the three aspects of offloading, task assignment and resource allocation simultaneously, and thus brings balanced improvement to all involved entities, and ultimately better system welfare.

### 5.3. Sensitivity Analysis of GTGO

A sensitivity analysis was performed to assess the robustness of the proposed GTGO framework by performing the same experimental setup described in the main evaluation (N = 30) task vehicles. The analysis investigates the effects of some important optimization parameters on the system welfare, with all other parameters set to their default values.

**Fig 6:**
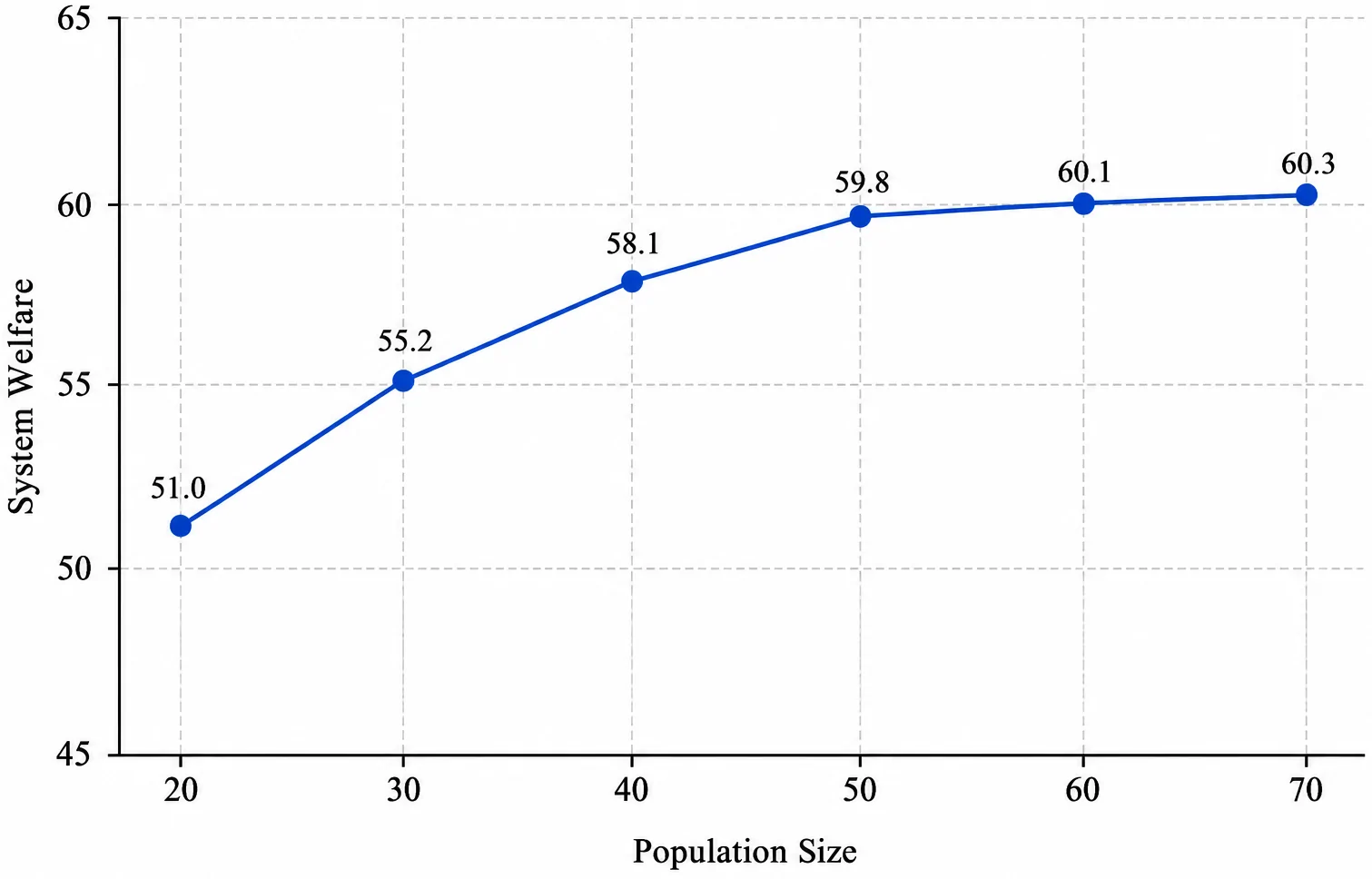
Effect of population size on system welfare (N = 30).

Fig 6 shows how system welfare is affected by population size. The welfare of the system increases markedly as the population size grows from 20 to 50, because of the better exploration of the search space and diversity of solutions obtained. When the population size is larger than 50, however, the improvement is very small and the cost of the computation increases. Thus, 50 is a good size for the population that will give a good balance of optimization quality and computational efficiency.

**Fig 7:**
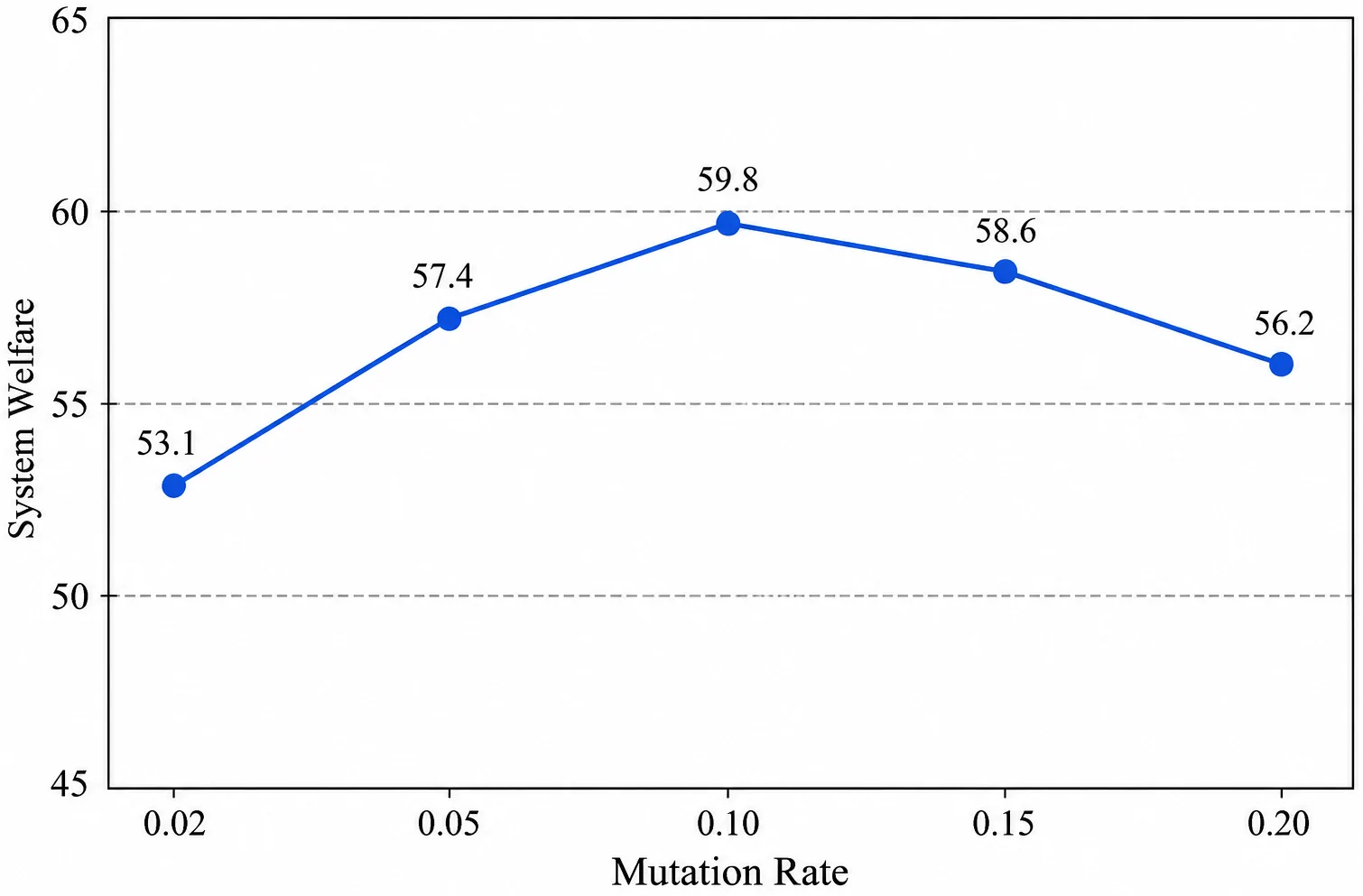
Effect of mutation rate on system welfare (N = 30).

The effect of mutation rate on system welfare is given in Fig 7. The outcomes demonstrate that system welfare starts off to rise with rising mutation rate but peaks at a mutation rate of 0.1. This behaviour is a sign of a successful exploration/exploitation trade-off in the evolutionary search process. A lower mutation rate might cause premature convergence, while a higher mutation rate may not allow for potentially good solutions to be disrupted and can negatively impact optimization results. In this way, a mutation rate of 0.1 was chosen as the default in the proposed GTGO framework.Overall, the sensitivity analysis confirms that GTGO is stable in its performance in a certain range of parameter values and is robust to the principle evolutionary optimization parameters (Table 4).

**Table 4:** Impact of XAI on Optimization Performance (*N* = 30).

Metric	GTGO + XAI	GTGO without XAI
System Welfare	59.8	59.4
Convergence Generation	60	58
Execution Time (s)	4.8	4.4
Interpretability	High	None

### 5.4. Impact of explainability on optimization performance

To assess the impact of the Explainable Artificial Intelligence (XAI) module on optimization performance, an ablation study was performed by comparing the full framework of GTGO (GTGO-XAI) to GTGO without the XAI module. The two designs were tested with the same simulation parameters using N = 30 task vehicles. The comparison of the proposed GTGO framework with and without the XAI module, under the same experimental conditions (N = 30), is shown in 4. The results presented that introducing explainability adds just a bit to the computational load, running the model takes 4.4 s while it takes 4.8 s when introduce explainability. The convergence behaviour is little different with both variants converging to stable solutions in about 60 generations. Moreover, the system welfare obtained by GTGO using XAI is marginally larger than the one obtained without XAI, suggesting that the effectiveness of the optimized system is not adversely impacted by the incorporation of explainability. The results show that transparency and interpretability is possible with negligible changes in computational speed and convergence properties.

## 6. Conclusion and future scope

This paper introduced a GTGO-based model of joint task offloading, scheduling, and resource allocation in a vehicular edge computing system, with an augmented integrated explainable AI (XAI) module to enhance the transparency of decisions. The proposed scheme is effective in modeling the interaction between the task vehicles, helper vehicles, and the edge nodes within the same system welfare goal and meeting the latency requirements. The experimental results show that the framework attains better system welfare with increasing number of task vehicles, and controlled average task delay in comparison to baseline methods. Moreover, the convergence analysis proves that the GTGO algorithm quickly reaches high-quality solutions and, as a result, there is an effective balance between exploration and exploitation in the optimization process. The XAI module adds further interpretability to the model, quantifying the contribution of key decision variables, thus enhancing trust and usability of the model in practice deployments. By and large, the suggested approach is scalable and transparent solution to smart resource management in dynamic vehicular edge computing issues.

Although the proposed approach was evaluated against Greedy Offloading, Local-Only Processing, and Random Assignment strategies,some recent studies have demonstrated the effectiveness of advanced optimization techniques such as Deep Reinforcement Learning (DRL) and Particle Swarm Optimization (PSO) for task offloading and resource allocation in vehicular edge computing environments. DRL-based methods can dynamically adapt to changing network conditions, while PSO-based approaches provide efficient population-based search capabilities. A comprehensive experimental comparison between GTGO and these advanced optimization techniques is an important direction for future research and will be considered in subsequent work.

The proposed GTGO-XAI framework can be adopted in next-generation Intelligent Transportation Systems (ITS) for real-time task offloading, resource allocation, and decision transparency within connected vehicular environments from a practical deployment perspective. For autonomous vehicle systems, the framework can help manage complex applications like object detection, trajectory planning, cooperative perception, and collision avoidance by effectively allocating compute resources between individual vehicles, roadside infrastructure and edge computing servers. Moreover, the explainability aspect can help in boosting the trustworthiness and regulatory adherence of the users, as it can offer understandable explanations for important resource allocation and offloading choices. The proposed framework can be scaled to support highly dynamic and data-intensive vehicular applications, while also being an intelligent management solution for 6G-enabled vehicular networks, which are anticipated to be ultra-low latency, with a massive number of connections and integrated AI services.

Further investigation can be aimed at applying the framework to the dynamic and large scale vehicular setting by considering mobility-aware and distributed optimization solutions. Besides, adaptability and interpretability in real-world applications can be further enhanced by integrating advanced learning methods like deep reinforcement learning and improving the XAI module to be richer and context-aware.
