# Supplementary material for: GTGO-driven joint task offloading and resource allocation with explainable AI in vehicular edge computing
Source: PLoS One. 2026 Aug 19;21(8):e0354977. doi: 10.1371/journal.pone.0354977 (PMC13489482; doi:10.1371/journal.pone.0354977)
Supplement: Supplementary file 1 [file pone.0354977.s002.docx]

**Reviewer 1**

**Comment 1:** The manuscript introduces a GTGO-based optimization framework; however, the distinction between GTGO and traditional Genetic Algorithm-based optimization is not clearly established.

**Response:**

We thank the reviewer for this insightful comment. To clarify the novelty of the proposed framework, a new subsection entitled **"Algorithmic Innovations of GTGO"** has been added in Section 4.5.1. The revised manuscript now explicitly distinguishes GTGO from conventional Genetic Algorithms through (i) joint multi-dimensional chromosome encoding, (ii) constraint-aware fitness evaluation, (iii) explainability-guided decision assessment. Additionally, a comparison table between conventional GA and GTGO has been included to highlight the proposed enhancements and their suitability for vehicular edge computing environments.

**Comment 2**

The paper presents the optimization framework and reports promising performance results, but no computational complexity analysis is provided.

**Response**

We thank the reviewer for this valuable suggestion. A new subsubsection entitled **“Computational Complexity Analysis”** has been added. The revised manuscript now derives the computational complexity of the proposed GTGO framework as $O(GPN)$, where $G$denotes the number of generations, $P$the population size, and $N$the number of task vehicles. Furthermore, a scalability discussion has been incorporated to demonstrate that the algorithm exhibits linear growth with respect to the number of task vehicles and converges within 30–50 generations in practical scenarios. The suitability of the framework for real-time vehicular edge computing deployments and its potential for parallel implementation on edge servers have also been discussed.

**Comment 3**

Although the integration of Explainable AI is one of the main contributions of the paper, the implementation details remain relatively abstract. The manuscript should provide a more rigorous explanation of how explanation scores are generated, how feature contributions are computed, and how explainability information influences the optimization process.

**Response**

We thank the reviewer for this valuable comment. To improve the clarity of the explainability component, Section 4.5.1 has been expanded with additional implementation details. The revised manuscript now explains the generation of feature contribution scores, the set of features considered in the analysis (task size, computational demand, delay threshold, resource availability, offloading ratio, and edge resource allocation), and the computation of the aggregated explainability score. Furthermore, we clarify that the explainability module operates as a post-optimization analysis mechanism that provides interpretable insights into GTGO decisions without altering the optimization process itself.

**Comment 4: The proposed method is compared with Greedy Offloading, Local-Only Processing, and Random Assignment approaches. However, these baselines are relatively simple and may not adequately demonstrate the superiority of the proposed framework. Comparisons with recent DRL-based and PSO-based resource allocation techniques would strengthen the experimental evaluation.**

**Response:** We thank the reviewer for this valuable suggestion. We agree that comparisons with advanced optimization techniques such as DRL-based and PSO-based resource allocation approaches would further strengthen the experimental evaluation. The current study primarily focuses on establishing the effectiveness of the proposed GTGO framework against widely used benchmark strategies, including Greedy Offloading, Local-Only Processing, and Random Assignment. To address the reviewer’s concern, we have expanded the discussion section for DRL-based and PSO-based optimization methods. A comprehensive implementation and experimental comparison with these advanced approaches has been identified as an important direction for future work.

**Comment 5: The discussion primarily focuses on performance improvements but provides limited insight into the reasons behind the observed gains. The authors should provide a deeper analysis explaining why GTGO achieves better welfare and lower delay compared to competing approaches and discuss the trade-offs involved.**

**Response:** Thankyou for this comment. To address this, I have added a new subsection in the result section and a graph addressing System Welfare Component Breakdown (N = 30) to provide additional insight into the sources of welfare improvement. The figure illustrates the contributions of task vehicles, helper vehicles, and the controller to the overall system welfare, demonstrating that GTGO achieves balanced gains across all participating entities.

**Comment 6: Several important parameters, such as mutation rate, population size, crossover rate, and resource availability, directly influence optimization performance. A sensitivity analysis examining the effect of these parameters on convergence speed and system welfare would provide valuable insights into the behavior of the proposed framework.**

**Response:** We thank the reviewer for this valuable suggestion. To address this comment two figures namely “Effect of population size on system welfare (N = 30)” and “Effect of mutation rate on system welfare (N = 30)” have been added into the result section and the explanation of the figures have been given in subsection “Sensitivity Analysis of GTGO”.

**Comment 7: The explainability is presented as a key contribution of the proposed framework, the manuscript does not quantitatively evaluate its effect on optimization performance. A comparative analysis between the GTGO framework with and without the XAI module would help determine whether transparency is achieved at the cost of increased computational overhead, slower convergence, or reduced optimization effectiveness.**

**Response:** Thankyou for the comment. A new subsection “Impact of Explainability on Optimization Performance” has been added in the result section in which a table compares various parameters when implemented GTGO+XAI and GTGO-XAI.

**Comment 8: The authors may consider referring to and citing the recent work “RLDJ-W: A Reinforcement Learning-driven Joint Watermarking Framework for Privacy Leakage Detection in Digital Healthcare Systems”. This study provides a reinforcement learning-driven framework related to privacy-aware intelligent optimization and security protection in digital systems. Discussing this work could help strengthen the manuscript’s background on learning-based decision-making frameworks, privacy protection, and intelligent resource, security optimization, and may provide useful comparative or methodological insights for the proposed GTGO-based framework.**

**Response:** Thank you for this valuable recommendation. We have reviewed the suggested work, *“RLDJ-W: A Reinforcement Learning-driven Joint Watermarking Framework for Privacy Leakage Detection in Digital Healthcare Systems,”* and incorporated it into the Related Work section of the revised manuscript.

**Reviewer 2**

**Comment 1: The introduction provides a comprehensive overview of vehicular edge computing, explainable AI, and task offloading. However, the specific research gap addressed by the proposed GTGO-XAI framework is not sufficiently highlighted. The authors should explicitly explain how existing approaches fail to jointly address offloading, resource allocation, and explainability, and clearly state the unique contributions of the proposed framework.**

**Response:**

Thank you for this valuable observation. We agree that the research gap and novelty of the proposed framework should be more explicitly articulated. To address this concern, we have revised the Introduction section by adding a dedicated discussion of the limitations of existing task offloading and resource allocation approaches in vehicular edge computing. The revised Introduction now clearly explains how the proposed GTGO-XAI framework fills this gap through the integration of GTGO-based optimization and explainable decision-making mechanisms.

**Comment 2: The literature review covers a broad range of studies related to federated learning, XAI, and vehicular edge computing. However, it lacks a structured comparison of existing approaches. A summary table comparing prior methods, optimization techniques, explainability mechanisms, advantages, and limitations would significantly improve readability and help readers understand the motivation behind the proposed work.**

**Response:** Thank you for this valuable suggestion. To improve the clarity and readability of the literature review, we have added a new comparative summary table in the Related Work section. The table systematically compares representative existing approaches with respect to optimization techniques, explainability mechanisms, key advantages, and limitations.

**Comment 3: The manuscript contains several architectural and workflow figures, but the associated descriptions are relatively brief. The authors should provide more detailed explanations of the information flow between layers, the role of each module, and how the optimization and explainability components interact within the proposed framework.**

**Response:** Thank you for this valuable suggestion. To improve the clarity of the proposed architecture, we have significantly expanded the description associated with Figure 1. The revised manuscript now provides a detailed layer-wise explanation of the Vehicular Layer, Edge Layer, Intelligence and Explainability Layer, and Control Layer. In addition, the information flow between layers has been explicitly described, including task generation, resource collection, optimization, decision dissemination, and execution processes.

**Comment 4: Some mathematical symbols and variables are introduced without sufficient explanation. In addition, certain variables appear in equations before being formally defined. The manuscript would benefit from a notation table summarizing all symbols, parameters, and decision variables used throughout the optimization framework.**

**Response:** Thank you for this valuable suggestion. We agree that the large number of symbols, parameters, and decision variables used throughout the optimization framework may affect readability. To improve clarity and consistency, we have added a new notation table that summarizes all major symbols, parameters, utility terms, and optimization decision variables used in the manuscript.

**Comment 5: Conclusion summarizes the findings, it provides limited discussion regarding real-world deployment. The authors should elaborate on how the proposed framework can be integrated into intelligent transportation systems, autonomous vehicle ecosystems, and future 6G-enabled vehicular networks.**

**Response:** Thank you for this valuable suggestion. To improve the practical relevance of the manuscript, we have expanded the Conclusion section by discussing potential real-world deployment scenarios of the proposed GTGO-XAI framework. Specifically, we describe how the framework can be integrated into Intelligent Transportation Systems (ITS), autonomous vehicle ecosystems, and future 6G-enabled vehicular networks. These additions provide a clearer perspective on the practical significance and future deployment potential of the proposed approach.
