# Supplementary material for: GTGO-driven joint task offloading and resource allocation with explainable AI in vehicular edge computing
Source: PLoS One. 2026 Aug 19;21(8):e0354977. doi: 10.1371/journal.pone.0354977 (PMC13489482; doi:10.1371/journal.pone.0354977)
Supplement: Supplementary file 2 [file pone.0354977.s003.docx]

**Response Sheet**

**Comment 1: The manuscript presents an interesting optimization framework; however, the novelty of the proposed GTGO-XAI approach should be stated more explicitly at the end of the Introduction. A concise list of the main contributions would help readers better understand the unique aspects of the proposed work.**

**Response:** Thank you for your valuable suggestion. We have revised the end of the Introduction by explicitly highlighting the novelty of the proposed GTGO-XAI framework and adding concisely the main contributions. The revised manuscript now clearly emphasizes the unique aspects of the proposed approach.

**Comment 2: Please ensure consistent use of terminology throughout the manuscript. For example, terms such as "system welfare," "overall welfare" and "social welfare" should be used consistently.**

**Response:** Thank you for your valuable observation. We have carefully reviewed the manuscript and standardized the terminology throughout. The term "system welfare" has been adopted consistently as the primary term to describe the optimization objective. All occurrences of terms such as "overall welfare" and "social welfare" have been revised to "system welfare" (or "overall system welfare" where context required) to ensure consistency and improve the clarity and readability of the manuscript.

**Comment 3:** Please verify that all variables, parameters and axes include appropriate units wherever applicable.

**Response:** Thank you for your careful observation. We have reviewed the manuscript to ensure that all physical variables, parameters, tables, and figure axes include appropriate units wherever applicable. The notation table has been updated to specify units for parameters such as input data size (MB), computational demand (MIPS), delay threshold (s), helper computational resources (MIPS).

**Comment 4:** Please carefully verify that all references follow the journal formatting guidelines and that citation styles are consistent throughout the manuscript.

**Response:** Thank you for your valuable suggestion. We have carefully reviewed the entire reference list and the in-text citations to ensure compliance with the journal's formatting guidelines.

**Comment 5:** The manuscript is generally well organized; however, several grammatical and typographical errors are present throughout the paper. A careful proofreading would improve the overall readability and presentation quality.

**Response:** Thank you for your constructive comment. We have carefully proofread the entire manuscript and corrected grammatical, typographical, punctuation, and language inconsistencies throughout the paper.
