# Supplementary material for: GTGO-driven joint task offloading and resource allocation with explainable AI in vehicular edge computing
Source: PLoS One. 2026 Aug 19;21(8):e0354977. doi: 10.1371/journal.pone.0354977 (PMC13489482; doi:10.1371/journal.pone.0354977)
Supplement: Peer review history

# Peer review: Decision Letter 0

**DOI:** 10.1371/journal.pone.0354977.r001

**Authors:** Muhammad Shahid Anwar

7 Jun 2026

Dear Dr. Khan,

Thank you for submitting your manuscript to PLOS ONE. After careful consideration, we feel that it has merit but does not fully meet PLOS ONE’s publication criteria as it currently stands. Therefore, we invite you to submit a revised version of the manuscript that addresses the points raised during the review process.

Please submit your revised manuscript by Aug 06 2026 11:59PM. If you will need more time than this to complete your revisions, please reply to this message or contact the journal office at plosone@plos.org. When you're ready to submit your revision, log on to https://www.editorialmanager.com/pone/ and select the 'Submissions Needing Revision' folder to locate your manuscript file.

- A letter that responds to each point raised by the academic editor and reviewer(s). You should upload this letter as a separate file labeled 'Response to Reviewers'.
- A marked-up copy of your manuscript that highlights changes made to the original version. You should upload this as a separate file labeled 'Revised Manuscript with Track Changes'.
- An unmarked version of your revised paper without tracked changes. You should upload this as a separate file labeled 'Manuscript'.

If you would like to make changes to your financial disclosure, please include your updated statement in your cover letter. Guidelines for resubmitting your figure files are available below the reviewer comments at the end of this letter.

If applicable, we recommend that you deposit your laboratory protocols in protocols.io to enhance the reproducibility of your results. Protocols.io assigns your protocol its own identifier (DOI) so that it can be cited independently in the future. For instructions see: https://journals.plos.org/plosone/s/submission-guidelines#loc-laboratory-protocols. Additionally, PLOS ONE offers an option for publishing peer-reviewed Lab Protocol articles, which describe protocols hosted on protocols.io. Read more information on sharing protocols at https://plos.org/protocols?utm_medium=editorial-email&utm_source=authorletters&utm_campaign=protocols.

As the corresponding author, your ORCID iD is verified in the submission system and will appear in the published article. PLOS supports the use of ORCID, and we encourage all coauthors to register for an ORCID iD and use it as well. Please encourage your coauthors to verify their ORCID iD within the submission system before final acceptance, as unverified ORCID iDs will not appear in the published article. *Only*  the individual author can complete the verification step; PLOS staff *cannot*  verify ORCID iDs on behalf of authors.

We look forward to receiving your revised manuscript.

Kind regards,

Muhammad Shahid Anwar

Academic Editor

PLOS One

Journal Requirements:

When submitting your revision, we need you to address these additional requirements.

1. Please ensure that your manuscript meets PLOS ONE's style requirements, including those for file naming. The PLOS ONE style templates can be found at

https://journals.plos.org/plosone/s/file?id=wjVg/PLOSOne_formatting_sample_main_body.pdf and

https://journals.plos.org/plosone/s/file?id=ba62/PLOSOne_formatting_sample_title_authors_affiliations.pdf

2. Please note that PLOS One has specific guidelines on code sharing for submissions in which author-generated code underpins the findings in the manuscript. In these cases, we expect all author-generated code to be made available without restrictions upon publication of the work. Please review our guidelines at https://journals.plos.org/plosone/s/materials-and-software-sharing#loc-sharing-code

and ensure that your code is shared in a way that follows best practice and facilitates reproducibility and reuse.

3. We note that the grant information you provided in the ‘Funding Information’ and ‘Financial Disclosure’ sections do not match.

When you resubmit, please ensure that you provide the correct grant numbers for the awards you received for your study in the ‘Funding Information’ section.

4. Thank you for stating the following in the Acknowledgments Section of your manuscript:

“The authors would like to thank University of Nottingham which has a MoU with PloS One”

We note that you have provided funding information that is not currently declared in your Funding Statement. However, funding information should not appear in the Acknowledgments section or other areas of your manuscript. We will only publish funding information present in the Funding Statement section of the online submission form.

Please remove any funding-related text from the manuscript and let us know how you would like to update your Funding Statement. Currently, your Funding Statement reads as follows:

“The author(s) received no specific funding for this work”

Please include your amended statements within your cover letter; we will change the online submission form on your behalf.

5. When completing the data availability statement of the submission form, you indicated that you will make your data available on acceptance. We strongly recommend all authors decide on a data sharing plan before acceptance, as the process can be lengthy and hold up publication timelines. Please note that, though access restrictions are acceptable now, your entire data will need to be made freely accessible if your manuscript is accepted for publication. This policy applies to all data except where public deposition would breach compliance with the protocol approved by your research ethics board. If you are unable to adhere to our open data policy, please kindly revise your statement to explain your reasoning and we will seek the editor's input on an exemption. Please be assured that, once you have provided your new statement, the assessment of your exemption will not hold up the peer review process.

6. Please upload a copy of Figure 1-4, to which you refer in your text on page 6, 10 and 11. If the figure is no longer to be included as part of the submission please remove all reference to it within the text.

7. We note you have included a table to which you do not refer in the text of your manuscript. Please ensure that you refer to Table 1 in your text; if accepted, production will need this reference to link the reader to the Table.

8. Please include captions for your Supporting Information files at the end of your manuscript, and update any in-text citations to match accordingly. Please see our Supporting Information guidelines for more information: http://journals.plos.org/plosone/s/supporting-information.

9. If the reviewer comments include a recommendation to cite specific previously published works, please review and evaluate these publications to determine whether they are relevant and should be cited. There is no requirement to cite these works unless the editor has indicated otherwise.

[Note: HTML markup is below. Please do not edit.]

Reviewers' comments:

Reviewer's Responses to Questions

**Comments to the Author**

1. Is the manuscript technically sound, and do the data support the conclusions?

Reviewer #1: Yes

Reviewer #2: Yes

**********

2. Has the statistical analysis been performed appropriately and rigorously?

Reviewer #1: Yes

Reviewer #2: Yes

**********

3. Have the authors made all data underlying the findings in their manuscript fully available?

The PLOS Data policy

Reviewer #1: Yes

Reviewer #2: Yes

**********

4. Is the manuscript presented in an intelligible fashion and written in standard English?

Reviewer #1: Yes

Reviewer #2: Yes

**********

Reviewer #1: Comment 1:

The manuscript introduces a GTGO-based optimization framework; however, the distinction between GTGO and traditional Genetic Algorithm-based optimization is not clearly established. The authors should provide a dedicated subsection explaining the algorithmic innovations, enhancements, or modifications introduced in GTGO and justify why it is expected to outperform existing evolutionary optimization methods.

Comment 2:

The paper presents the optimization framework and reports promising performance results, but no computational complexity analysis is provided. Since vehicular edge computing environments are highly dynamic and latency-sensitive, the authors should analyze the time complexity and scalability of the proposed GTGO algorithm and discuss its suitability for real-time deployment.

Comment 3:

Although the integration of Explainable AI is one of the main contributions of the paper, the implementation details remain relatively abstract. The manuscript should provide a more rigorous explanation of how explanation scores are generated, how feature contributions are computed, and how explainability information influences the optimization process.

Comment 4:

The proposed method is compared with Greedy Offloading, Local-Only Processing, and Random Assignment approaches. However, these baselines are relatively simple and may not adequately demonstrate the superiority of the proposed framework. Comparisons with recent DRL-based and PSO-based resource allocation techniques would strengthen the experimental evaluation.

Comment 5:

The discussion primarily focuses on performance improvements but provides limited insight into the reasons behind the observed gains. The authors should provide a deeper analysis explaining why GTGO achieves better welfare and lower delay compared to competing approaches and discuss the trade-offs involved.

Comment 6:

Several important parameters, such as mutation rate, population size, crossover rate, and resource availability, directly influence optimization performance. A sensitivity analysis examining the effect of these parameters on convergence speed and system welfare would provide valuable insights into the behavior of the proposed framework.

Comment 7:

The explainability is presented as a key contribution of the proposed framework, the manuscript does not quantitatively evaluate its effect on optimization performance. A comparative analysis between the GTGO framework with and without the XAI module would help determine whether transparency is achieved at the cost of increased computational overhead, slower convergence, or reduced optimization effectiveness.

Comment 8:

The authors may consider referring to and citing the recent work “RLDJ-W: A Reinforcement Learning-driven Joint Watermarking Framework for Privacy Leakage Detection in Digital Healthcare Systems”. This study provides a reinforcement learning-driven framework related to privacy-aware intelligent optimization and security protection in digital systems. Discussing this work could help strengthen the manuscript’s background on learning-based decision-making frameworks, privacy protection, and intelligent resource, security optimization, and may provide useful comparative or methodological insights for the proposed GTGO-based framework.

Reviewer #2: Comment 1:

The introduction provides a comprehensive overview of vehicular edge computing, explainable AI, and task offloading. However, the specific research gap addressed by the proposed GTGO-XAI framework is not sufficiently highlighted. The authors should explicitly explain how existing approaches fail to jointly address offloading, resource allocation, and explainability, and clearly state the unique contributions of the proposed framework.

Comment 2:

The literature review covers a broad range of studies related to federated learning, XAI, and vehicular edge computing. However, it lacks a structured comparison of existing approaches. A summary table comparing prior methods, optimization techniques, explainability mechanisms, advantages, and limitations would significantly improve readability and help readers understand the motivation behind the proposed work.

Comment 3:

The manuscript contains several architectural and workflow figures, but the associated descriptions are relatively brief. The authors should provide more detailed explanations of the information flow between layers, the role of each module, and how the optimization and explainability components interact within the proposed framework.

Comment 4:

Some mathematical symbols and variables are introduced without sufficient explanation. In addition, certain variables appear in equations before being formally defined. The manuscript would benefit from a notation table summarizing all symbols, parameters, and decision variables used throughout the optimization framework.

Comment 5:

Conclusion summarizes the findings, it provides limited discussion regarding real-world deployment. The authors should elaborate on how the proposed framework can be integrated into intelligent transportation systems, autonomous vehicle ecosystems, and future 6G-enabled vehicular networks.

**********

what does this mean?). If published, this will include your full peer review and any attached files.

If you choose “no”, your identity will remain anonymous but your review may still be made public.

**Do you want your identity to be public for this peer review?**  For information about this choice, including consent withdrawal, please see our Privacy Policy

Reviewer #1: No

Reviewer #2: No

**********

[NOTE: If reviewer comments were submitted as an attachment file, they will be attached to this email and accessible via the submission site. Please log into your account, locate the manuscript record, and check for the action link "View Attachments". If this link does not appear, there are no attachment files.]

To ensure your figures meet our technical requirements, please review our figure guidelines: https://journals.plos.org/plosone/s/figures

You may also use PLOS’s free figure tool, NAAS, to help you prepare publication quality figures: https://journals.plos.org/plosone/s/figures#loc-tools-for-figure-preparation.

NAAS will assess whether your figures meet our technical requirements by comparing each figure against our figure specifications.

# Author comment: Author response to Decision Letter 1

**DOI:** 10.1371/journal.pone.0354977.r002

2 Jul 2026

Reviewer 1

Comment 1: The manuscript introduces a GTGO-based optimization framework; however, the distinction between GTGO and traditional Genetic Algorithm-based optimization is not clearly established.

Response:

We thank the reviewer for this insightful comment. To clarify the novelty of the proposed framework, a new subsection entitled "Algorithmic Innovations of GTGO" has been added in Section 4.5.1. The revised manuscript now explicitly distinguishes GTGO from conventional Genetic Algorithms through (i) joint multi-dimensional chromosome encoding, (ii) constraint-aware fitness evaluation, (iii) explainability-guided decision assessment. Additionally, a comparison table between conventional GA and GTGO has been included to highlight the proposed enhancements and their suitability for vehicular edge computing environments.

Comment 2

The paper presents the optimization framework and reports promising performance results, but no computational complexity analysis is provided.

Response

We thank the reviewer for this valuable suggestion. A new subsubsection entitled “Computational Complexity Analysis” has been added. The revised manuscript now derives the computational complexity of the proposed GTGO framework as O(GPN), where Gdenotes the number of generations, Pthe population size, and Nthe number of task vehicles. Furthermore, a scalability discussion has been incorporated to demonstrate that the algorithm exhibits linear growth with respect to the number of task vehicles and converges within 30–50 generations in practical scenarios. The suitability of the framework for real-time vehicular edge computing deployments and its potential for parallel implementation on edge servers have also been discussed.

Comment 3

Although the integration of Explainable AI is one of the main contributions of the paper, the implementation details remain relatively abstract. The manuscript should provide a more rigorous explanation of how explanation scores are generated, how feature contributions are computed, and how explainability information influences the optimization process.

Response

We thank the reviewer for this valuable comment. To improve the clarity of the explainability component, Section 4.5.1 has been expanded with additional implementation details. The revised manuscript now explains the generation of feature contribution scores, the set of features considered in the analysis (task size, computational demand, delay threshold, resource availability, offloading ratio, and edge resource allocation), and the computation of the aggregated explainability score. Furthermore, we clarify that the explainability module operates as a post-optimization analysis mechanism that provides interpretable insights into GTGO decisions without altering the optimization process itself.

Comment 4: The proposed method is compared with Greedy Offloading, Local-Only Processing, and Random Assignment approaches. However, these baselines are relatively simple and may not adequately demonstrate the superiority of the proposed framework. Comparisons with recent DRL-based and PSO-based resource allocation techniques would strengthen the experimental evaluation.

Response: We thank the reviewer for this valuable suggestion. We agree that comparisons with advanced optimization techniques such as DRL-based and PSO-based resource allocation approaches would further strengthen the experimental evaluation. The current study primarily focuses on establishing the effectiveness of the proposed GTGO framework against widely used benchmark strategies, including Greedy Offloading, Local-Only Processing, and Random Assignment. To address the reviewer’s concern, we have expanded the discussion section for DRL-based and PSO-based optimization methods. A comprehensive implementation and experimental comparison with these advanced approaches has been identified as an important direction for future work.

Comment 5: The discussion primarily focuses on performance improvements but provides limited insight into the reasons behind the observed gains. The authors should provide a deeper analysis explaining why GTGO achieves better welfare and lower delay compared to competing approaches and discuss the trade-offs involved.

Response: Thankyou for this comment. To address this, I have added a new subsection in the result section and a graph addressing System Welfare Component Breakdown (N = 30) to provide additional insight into the sources of welfare improvement. The figure illustrates the contributions of task vehicles, helper vehicles, and the controller to the overall system welfare, demonstrating that GTGO achieves balanced gains across all participating entities.

Comment 6: Several important parameters, such as mutation rate, population size, crossover rate, and resource availability, directly influence optimization performance. A sensitivity analysis examining the effect of these parameters on convergence speed and system welfare would provide valuable insights into the behavior of the proposed framework.

Response: We thank the reviewer for this valuable suggestion. To address this comment two figures namely “Effect of population size on system welfare (N = 30)” and “Effect of mutation rate on system welfare (N = 30)” have been added into the result section and the explanation of the figures have been given in subsection “Sensitivity Analysis of GTGO”.

Comment 7: The explainability is presented as a key contribution of the proposed framework, the manuscript does not quantitatively evaluate its effect on optimization performance. A comparative analysis between the GTGO framework with and without the XAI module would help determine whether transparency is achieved at the cost of increased computational overhead, slower convergence, or reduced optimization effectiveness.

Response: Thankyou for the comment. A new subsection “Impact of Explainability on Optimization Performance” has been added in the result section in which a table compares various parameters when implemented GTGO+XAI and GTGO-XAI.

Comment 8: The authors may consider referring to and citing the recent work “RLDJ-W: A Reinforcement Learning-driven Joint Watermarking Framework for Privacy Leakage Detection in Digital Healthcare Systems”. This study provides a reinforcement learning-driven framework related to privacy-aware intelligent optimization and security protection in digital systems. Discussing this work could help strengthen the manuscript’s background on learning-based decision-making frameworks, privacy protection, and intelligent resource, security optimization, and may provide useful comparative or methodological insights for the proposed GTGO-based framework.

Response: Thank you for this valuable recommendation. We have reviewed the suggested work, “RLDJ-W: A Reinforcement Learning-driven Joint Watermarking Framework for Privacy Leakage Detection in Digital Healthcare Systems,” and incorporated it into the Related Work section of the revised manuscript.

Reviewer 2

Comment 1: The introduction provides a comprehensive overview of vehicular edge computing, explainable AI, and task offloading. However, the specific research gap addressed by the proposed GTGO-XAI framework is not sufficiently highlighted. The authors should explicitly explain how existing approaches fail to jointly address offloading, resource allocation, and explainability, and clearly state the unique contributions of the proposed framework.

Response:

Thank you for this valuable observation. We agree that the research gap and novelty of the proposed framework should be more explicitly articulated. To address this concern, we have revised the Introduction section by adding a dedicated discussion of the limitations of existing task offloading and resource allocation approaches in vehicular edge computing. The revised Introduction now clearly explains how the proposed GTGO-XAI framework fills this gap through the integration of GTGO-based optimization and explainable decision-making mechanisms.

Comment 2: The literature review covers a broad range of studies related to federated learning, XAI, and vehicular edge computing. However, it lacks a structured comparison of existing approaches. A summary table comparing prior methods, optimization techniques, explainability mechanisms, advantages, and limitations would significantly improve readability and help readers understand the motivation behind the proposed work.

Response: Thank you for this valuable suggestion. To improve the clarity and readability of the literature review, we have added a new comparative summary table in the Related Work section. The table systematically compares representative existing approaches with respect to optimization techniques, explainability mechanisms, key advantages, and limitations.

Comment 3: The manuscript contains several architectural and workflow figures, but the associated descriptions are relatively brief. The authors should provide more detailed explanations of the information flow between layers, the role of each module, and how the optimization and explainability components interact within the proposed framework.

Response: Thank you for this valuable suggestion. To improve the clarity of the proposed architecture, we have significantly expanded the description associated with Figure 1. The revised manuscript now provides a detailed layer-wise explanation of the Vehicular Layer, Edge Layer, Intelligence and Explainability Layer, and Control Layer. In addition, the information flow between layers has been explicitly described, including task generation, resource collection, optimization, decision dissemination, and execution processes.

Comment 4: Some mathematical symbols and variables are introduced without sufficient explanation. In addition, certain variables appear in equations before being formally defined. The manuscript would benefit from a notation table summarizing all symbols, parameters, and decision variables used throughout the optimization framework.

Response: Thank you for this valuable suggestion. We agree that the large number of symbols, parameters, and decision variables used throughout the optimization framework may affect readability. To improve clarity and consistency, we have added a new notation table that summarizes all major symbols, parameters, utility terms, and optimization decision variables used in the manuscript.

Comment 5: Conclusion summarizes the findings, it provides limited discussion regarding real-world deployment. The authors should elaborate on how the proposed framework can be integrated into intelligent transportation systems, autonomous vehicle ecosystems, and future 6G-enabled vehicular networks.

Response: Thank you for this valuable suggestion. To improve the practical relevance of the manuscript, we have expanded the Conclusion section by discussing potential real-world deployment scenarios of the proposed GTGO-XAI framework. Specifically, we describe how the framework can be integrated into Intelligent Transportation Systems (ITS), autonomous vehicle ecosystems, and future 6G-enabled vehicular networks. These additions provide a clearer perspective on the practical significance and future deployment potential of the proposed approach.

Attachment Submitted filename: Response sheet plos.docx

# Peer review: Decision Letter 1

**DOI:** 10.1371/journal.pone.0354977.r003

**Authors:** Muhammad Shahid Anwar

7 Jul 2026

Dear Dr. Khan,

Thank you for submitting your manuscript to PLOS ONE. After careful consideration, we feel that it has merit but does not fully meet PLOS ONE’s publication criteria as it currently stands. Therefore, we invite you to submit a revised version of the manuscript that addresses the points raised during the review process.

Please submit your revised manuscript by Aug 06 2026 11:59PM. If you will need more time than this to complete your revisions, please reply to this message or contact the journal office at plosone@plos.org. When you're ready to submit your revision, log on to https://www.editorialmanager.com/pone/ and select the 'Submissions Needing Revision' folder to locate your manuscript file.

- A letter that responds to each point raised by the academic editor and reviewer(s). You should upload this letter as a separate file labeled 'Response to Reviewers'.
- A marked-up copy of your manuscript that highlights changes made to the original version. You should upload this as a separate file labeled 'Revised Manuscript with Track Changes'.
- An unmarked version of your revised paper without tracked changes. You should upload this as a separate file labeled 'Manuscript'.

If you would like to make changes to your financial disclosure, please include your updated statement in your cover letter. Guidelines for resubmitting your figure files are available below the reviewer comments at the end of this letter.

If applicable, we recommend that you deposit your laboratory protocols in protocols.io to enhance the reproducibility of your results. Protocols.io assigns your protocol its own identifier (DOI) so that it can be cited independently in the future. For instructions see: https://journals.plos.org/plosone/s/submission-guidelines#loc-laboratory-protocols. Additionally, PLOS ONE offers an option for publishing peer-reviewed Lab Protocol articles, which describe protocols hosted on protocols.io. Read more information on sharing protocols at https://plos.org/protocols?utm_medium=editorial-email&utm_source=authorletters&utm_campaign=protocols.

As the corresponding author, your ORCID iD is verified in the submission system and will appear in the published article. PLOS supports the use of ORCID, and we encourage all coauthors to register for an ORCID iD and use it as well. Please encourage your coauthors to verify their ORCID iD within the submission system before final acceptance, as unverified ORCID iDs will not appear in the published article. *Only*  the individual author can complete the verification step; PLOS staff *cannot*  verify ORCID iDs on behalf of authors.

We look forward to receiving your revised manuscript.

Kind regards,

Muhammad Shahid Anwar

Academic Editor

PLOS One

Journal Requirements:

If the reviewer comments include a recommendation to cite specific previously published works, please review and evaluate these publications to determine whether they are relevant and should be cited. There is no requirement to cite these works unless the editor has indicated otherwise.

Please review your reference list to ensure that it is complete and correct. If you have cited papers that have been retracted, please include the rationale for doing so in the manuscript text, or remove these references and replace them with relevant current references. Any changes to the reference list should be mentioned in the rebuttal letter that accompanies your revised manuscript. If you need to cite a retracted article, indicate the article’s retracted status in the References list and also include a citation and full reference for the retraction notice.

Additional Editor Comments:

Minor revision required

[Note: HTML markup is below. Please do not edit.]

Reviewers' comments:

Reviewer's Responses to Questions

**Comments to the Author**

Reviewer #1: All comments have been addressed

Reviewer #2: All comments have been addressed

**********

2. Is the manuscript technically sound, and do the data support the conclusions?

Reviewer #1: Yes

Reviewer #2: Yes

**********

3. Has the statistical analysis been performed appropriately and rigorously?

Reviewer #1: Yes

Reviewer #2: Yes

**********

4. Have the authors made all data underlying the findings in their manuscript fully available?

The PLOS Data policy

Reviewer #1: Yes

Reviewer #2: Yes

**********

5. Is the manuscript presented in an intelligible fashion and written in standard English?

Reviewer #1: Yes

Reviewer #2: Yes

**********

Reviewer #1: (No Response)

Reviewer #2: 1.The manuscript presents an interesting optimization framework; however, the novelty of the proposed GTGO-XAI approach should be stated more explicitly at the end of the Introduction. A concise list of the main contributions would help readers better understand the unique aspects of the proposed work.

2.Please ensure consistent use of terminology throughout the manuscript. For example, terms such as "system welfare," "overall welfare" and "social welfare" should be used consistently.

3.Please verify that all variables, parameters and axes include appropriate units wherever applicable.

4.Please carefully verify that all references follow the journal formatting guidelines and that citation styles are consistent throughout the manuscript.

5.The manuscript is generally well organized; however, several grammatical and typographical errors are present throughout the paper. A careful proofreading would improve the overall readability and presentation quality.

**********

what does this mean?). If published, this will include your full peer review and any attached files.

If you choose “no”, your identity will remain anonymous but your review may still be made public.

**Do you want your identity to be public for this peer review?**  For information about this choice, including consent withdrawal, please see our Privacy Policy

Reviewer #1: No

Reviewer #2: **Yes:** Imran

**********

[NOTE: If reviewer comments were submitted as an attachment file, they will be attached to this email and accessible via the submission site. Please log into your account, locate the manuscript record, and check for the action link "View Attachments". If this link does not appear, there are no attachment files.]

To ensure your figures meet our technical requirements, please review our figure guidelines: https://journals.plos.org/plosone/s/figures

You may also use PLOS’s free figure tool, NAAS, to help you prepare publication quality figures: https://journals.plos.org/plosone/s/figures#loc-tools-for-figure-preparation.

NAAS will assess whether your figures meet our technical requirements by comparing each figure against our figure specifications.

# Author comment: Author response to Decision Letter 2

**DOI:** 10.1371/journal.pone.0354977.r004

10 Jul 2026

Response Sheet

Comment 1: The manuscript presents an interesting optimization framework; however, the novelty of the proposed GTGO-XAI approach should be stated more explicitly at the end of the Introduction. A concise list of the main contributions would help readers better understand the unique aspects of the proposed work.

Response: Thank you for your valuable suggestion. We have revised the end of the Introduction by explicitly highlighting the novelty of the proposed GTGO-XAI framework and adding concisely the main contributions. The revised manuscript now clearly emphasizes the unique aspects of the proposed approach.

Comment 2: Please ensure consistent use of terminology throughout the manuscript. For example, terms such as "system welfare," "overall welfare" and "social welfare" should be used consistently.

Response: Thank you for your valuable observation. We have carefully reviewed the manuscript and standardized the terminology throughout. The term "system welfare" has been adopted consistently as the primary term to describe the optimization objective. All occurrences of terms such as "overall welfare" and "social welfare" have been revised to "system welfare" (or "overall system welfare" where context required) to ensure consistency and improve the clarity and readability of the manuscript.

Comment 3: Please verify that all variables, parameters and axes include appropriate units wherever applicable.

Response: Thank you for your careful observation. We have reviewed the manuscript to ensure that all physical variables, parameters, tables, and figure axes include appropriate units wherever applicable. The notation table has been updated to specify units for parameters such as input data size (MB), computational demand (MIPS), delay threshold (s), helper computational resources (MIPS).

Comment 4: Please carefully verify that all references follow the journal formatting guidelines and that citation styles are consistent throughout the manuscript.

Response: Thank you for your valuable suggestion. We have carefully reviewed the entire reference list and the in-text citations to ensure compliance with the journal's formatting guidelines.

Comment 5: The manuscript is generally well organized; however, several grammatical and typographical errors are present throughout the paper. A careful proofreading would improve the overall readability and presentation quality.

Response: Thank you for your constructive comment. We have carefully proofread the entire manuscript and corrected grammatical, typographical, punctuation, and language inconsistencies throughout the paper.

Attachment Submitted filename: Minor response sheet.docx

# Peer review: Decision Letter 2

**DOI:** 10.1371/journal.pone.0354977.r005

**Authors:** Muhammad Shahid Anwar

16 Jul 2026

GTGO-Driven Joint Task Offloading and Resource Allocation with Explainable AI in Vehicular Edge Computing

PONE-D-26-24293R2

Dear Dr. Khan,

We’re pleased to inform you that your manuscript has been judged scientifically suitable for publication and will be formally accepted for publication once it meets all outstanding technical requirements.

Within one week, you’ll receive an e-mail detailing the required amendments. When these have been addressed, you’ll receive a formal acceptance letter and your manuscript will be scheduled for publication.

An invoice will be generated when your article is formally accepted. Please note, if your institution has a publishing partnership with PLOS and your article meets the relevant criteria, all or part of your publication costs will be covered. Please make sure your user information is up-to-date by logging into Editorial Manager at Editorial Manager® and clicking the ‘Update My Information' link at the top of the page. For questions related to billing, please contact billing support.

If your institution or institutions have a press office, please notify them about your upcoming paper to help maximize its impact. If they’ll be preparing press materials, please inform our press team as soon as possible -- no later than 48 hours after receiving the formal acceptance. Your manuscript will remain under strict press embargo until 2 pm Eastern Time on the date of publication. For more information, please contact onepress@plos.org.

Kind regards,

Muhammad Shahid Anwar

Academic Editor

PLOS One

Additional Editor Comments (optional):

Reviewers' comments:

Reviewer's Responses to Questions

**Comments to the Author**

Reviewer #2: All comments have been addressed

**********

2. Is the manuscript technically sound, and do the data support the conclusions?

Reviewer #2: Yes

**********

3. Has the statistical analysis been performed appropriately and rigorously?

Reviewer #2: Yes

**********

4. Have the authors made all data underlying the findings in their manuscript fully available?

The PLOS Data policy

Reviewer #2: Yes

**********

5. Is the manuscript presented in an intelligible fashion and written in standard English?

Reviewer #2: (No Response)

**********

Reviewer #2: All comments are addressed accept for publication

Author well organised their manuscript, I recommend for publication

**********

what does this mean?). If published, this will include your full peer review and any attached files.

If you choose “no”, your identity will remain anonymous but your review may still be made public.

**Do you want your identity to be public for this peer review?**  For information about this choice, including consent withdrawal, please see our Privacy Policy

Reviewer #2: **Yes:** Imran

**********

# Editor report: Acceptance letter

**DOI:** 10.1371/journal.pone.0354977.r006

**Authors:** Muhammad Shahid Anwar

PONE-D-26-24293R2

PLOS One

Dear Dr. Khan,

I'm pleased to inform you that your manuscript has been deemed suitable for publication in PLOS One. Congratulations! Your manuscript is now being handed over to our production team.

At this stage, our production department will prepare your paper for publication. This includes ensuring the following:

* All references, tables, and figures are properly cited

* All relevant supporting information is included in the manuscript submission,

* There are no issues that prevent the paper from being properly typeset

You will receive further instructions from the production team, including instructions on how to review your proof when it is ready. Please keep in mind that we are working through a large volume of accepted articles, so please give us a few days to review your paper and let you know the next and final steps.

Lastly, if your institution or institutions have a press office, please let them know about your upcoming paper now to help maximize its impact. If they'll be preparing press materials, please inform our press team within the next 48 hours. Your manuscript will remain under strict press embargo until 2 pm Eastern Time on the date of publication. For more information, please contact onepress@plos.org.

You will receive an invoice from PLOS for your publication fee after your manuscript has reached the completed accept phase. If you receive an email requesting payment before acceptance or for any other service, this may be a phishing scheme. Learn how to identify phishing emails and protect your accounts at https://explore.plos.org/phishing.

If we can help with anything else, please email us at customercare@plos.org.

Thank you for submitting your work to PLOS ONE and supporting open access.

Kind regards,

PLOS ONE Editorial Office Staff

on behalf of

Professor Muhammad Shahid Anwar

Academic Editor

PLOS One
